# The novel anti-androgen candidate galeterone targets deubiquitinating enzymes, USP12 and USP46, to control prostate cancer growth and survival

**DOI:** 10.18632/oncotarget.25167

**Published:** 2018-05-18

**Authors:** Urszula L. McClurg, Mahsa Azizyan, Daniel T. Dransfield, Nivedita Namdev, Nay C.T.H. Chit, Sirintra Nakjang, Craig N. Robson

**Affiliations:** ^1^ Northern Institute for Cancer Research, Newcastle University, Newcastle upon Tyne, NE2 4HH, UK; ^2^ Tokai Pharmaceuticals, 255 State Street, Boston, MA 02109, USA; ^3^ Bioinformatics Support Unit, Faculty of Medical Sciences, Newcastle University, Newcastle upon Tyne, NE2 4HH, UK; ^4^ Institute for Cell and Molecular Biosciences, Medical School, Newcastle University, Newcastle upon Tyne, NE2 4HH, UK; ^5^ Current address: Siamab Therapeutics, Suite 100, Newton, MA 02458, USA

**Keywords:** prostate cancer, castrate-resistance, USP12, USP46, galeterone

## Abstract

Metastatic castration resistant prostate cancer is one of the main causes of male cancer associated deaths worldwide. Development of resistance is inevitable in patients treated with anti-androgen therapies. This highlights a need for novel therapeutic strategies that would be aimed upstream of the androgen receptor (AR). Here we report that the novel small molecule anti-androgen, galeterone targets USP12 and USP46, two highly homologous deubiquitinating enzymes that control the AR-AKT-MDM2-P53 signalling pathway. Consequently, galeterone is effective in multiple models of prostate cancer including both castrate resistant and AR-negative prostate cancer. However, we have observed that USP12 and USP46 selectively regulate full length AR protein but not the AR variants. This is the first report of deubiquitinating enzyme targeting as a strategy in prostate cancer treatment which we show to be effective in multiple, currently incurable models of this disease.

## INTRODUCTION

Prostate cancer (PC) is one of the main causes of cancer-related mortality in men worldwide. In the early stages of the disease patients respond well to anti-androgen based therapies however, resistance invariably develops within approximately two years. Even in castrate resistant PC (CRPC) the androgen receptor (AR) signalling cascade remains the critical pathway. Currently, multiple novel second line anti-androgens have been discovered with abiraterone and MDV3100 (enzalutamide) introduced into the clinical regime. However, these therapeutics offer only a short life expectancy advantage and resistance to them is still inevitable [1]. Additionally, patients who express constitutively active AR variants (AR Vs) lacking the ligand binding domain are intrinsically resistant to these novel treatments similarly to patients who lose AR expression and rely on alternative pathways [2, 3]. This highlights the need for developing novel compounds that could target all stages of PC.

Galeterone is a first-in-class multi-target oral small molecule with three reported mechanisms of activity; CYP17 lyase inhibition, AR antagonism, and induction of AR degradation [4–6]. Galeterone has shown significant anti-tumour activity with a well-tolerated safety profile in patients with CRPC in phase I and II clinical studies [7] including in six out of seven Phase II patients who were identified in a retrospective subset analysis as having truncated AR with *C*-terminal loss [7]. Previous *in vitro* studies demonstrated galeterone to be effective in cell lines with AR variant expression [8]. It was hypothesised that AR degradation, including degradation of the flutamide-resistant mutant AR T878A, stimulated by galeterone occurs via MDM2/CHIP activity [8, 9]. An evaluation of the kinetics of galeterone mediated degradation of AR demonstrated that AR V7 degradation requires substantially longer exposure to galeterone than AR FL. Additionally, galeterone was demonstrated to be effective against AR-negative prostate cell lines, this was proposed to be a consequence of eIF2α phosphorylation, antagonising the Mnk-eIF4E axis, and NF-κB and Twist1 inhibition [10]. Further studies have shown that abiraterone remains highly effective following galeterone treatment whereas enzalutamide and chemotherapy showed limited efficiency [11]. However, it was recently reported that galeterone and abiraterone might both be able to replace cholesterol as a substrate in cholesterolysis resulting in covalent hedgehog-drug conjugates activating hedgehog signalling *in vitro*, if this was also true *in vivo* it could stimulate PC progression in patients treated with galeterone [12].

USP12 and USP46 were initially identified as deubiquitinases (DUBs) targeting H2A and H2B in frog oocytes [13]. They are highly homologous sharing 89% sequence identity at the protein level, with only 11 amino acids in the whole sequence showing no degree of conservation, and contain a conserved bipartite nuclear localisation sequence (Supplementary Figure 1). Both enzymes require the WD40 protein, UAF1 for enzymatic activity with WDR20 further required to achieve the full enzymatic potential [14–19]. Since their identification, both proteins have been reported to regulate the AKT phosphatases, PHLPP and PHLPPL [20–22, 62], affect stabilisation of the cell surface T-cell receptor via deubiquitination of LAT and Trat1 [23] and to regulate the immune response following exposure to the Epstein-Barr virus [24]. Additionally, USP12 has been linked to the deubiquitination of non-activated Notch resulting in negative regulation of the Notch pathway [25] and regulation of immunity responses to LPS [26]. In contrast, USP46 has been reported to play a role in the nervous system by deubiquitination of AMPA [27] and GLR-1 [28]. Consequently, USP46 mutations and deletions affect behaviour in mice [29–32]. In humans, USP46 has also been linked to the development of depression and schizophrenia [33, 34].

As galeterone was shown to regulate AR and MDM2 stability through an unknown mechanism, in this manuscript we have focused on investigating the effects of galeterone on USP12 and USP46 deubiquitinating enzymes. We were able to identify a significant functional overlap between these two enzymes which is not surprising considering their high degree of homology. Additionally, we found that as well as inhibiting CYP17 and antagonising AR, galeterone is able to inhibit USP12 and USP46 enzymatic activity towards their targets including the AR and P53 pathway. Our data demonstrates a proof-of-principle for USP12 and USP46 complex targeting in PC and uncovers additional mechanisms of galeterone activity.

## RESULTS

### Galeterone binds to USP12 and USP46

**Figure 1:**
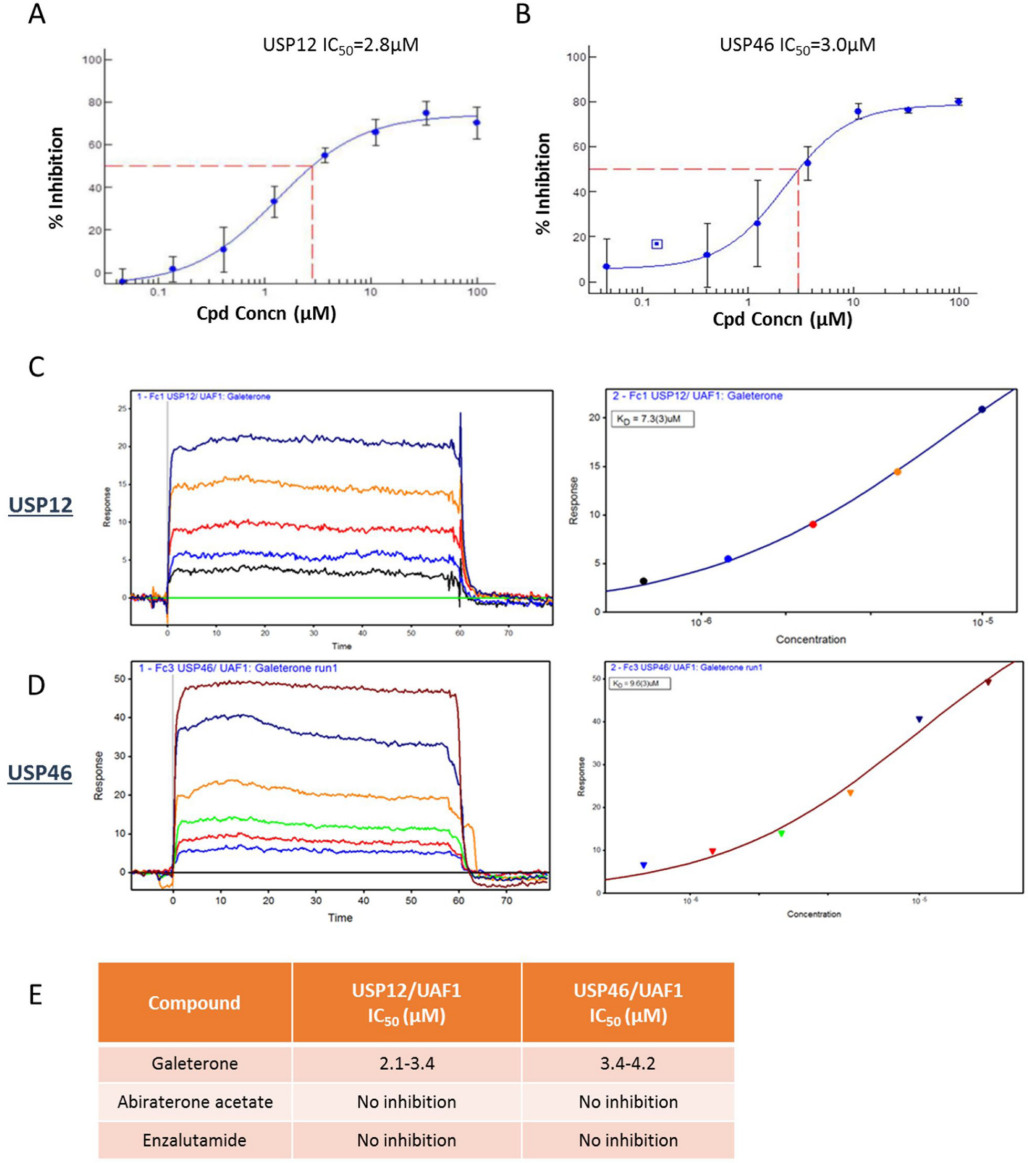
Galeterone binds USP12 and USP46 (**A**–**B**) IC_50_ curve fit values for inhibition of USP12/UAF1 (A) and USP46/UAF1 (B) generated using the XLFit program with a 4-parameter logistical fit. Ub-AMC was used as a substrate in the enzyme inhibition reaction (generated by Boston Biochem). (**C**–**D**) SPR binding data using Biacore. (E) IC_50_ comparison for novel antiandrogens.

It has been previously reported that galeterone affects AR protein stability. In prostate cells AR protein levels are regulated by an interplay between E3 ubiquitin ligases and deubiquitinating enzymes with USP10 [35, 36], USP12 [37] and USP26 [38] previously reported to deubiquitinate the AR. To investigate the mechanism behind galeterone's regulation of AR protein stability we screened a panel of enzymatically active deubiquitinating enzymes *in vitro* which demonstrated that galeterone selectively inhibited the enzymatic activity of only two DUBs USP12 (IC_50_ 2.1-3.4 μM) and USP46 (IC_50_ 3.4-4.2 μM) (Figure 1A). BIAcore SPR studies demonstrated a dose dependent binding of galeterone to USP12 and USP46 (Figure 1C, 1D). It is unsurprising that both of these enzymes were identified considering their high homology (Supplementary Figure 1), shared yeast orthologue Ubp9 [39] and the same interacting partners UAF1 and WDR20 [14, 15, 18, 40]. Additionally silencing either USP12 or USP46 expression has similar effects on pAKT levels, consistent with their role in deubiquitination of PHLPPs [20, 21].

### USP12 and USP46 have a functional overlap

**Figure 2:**
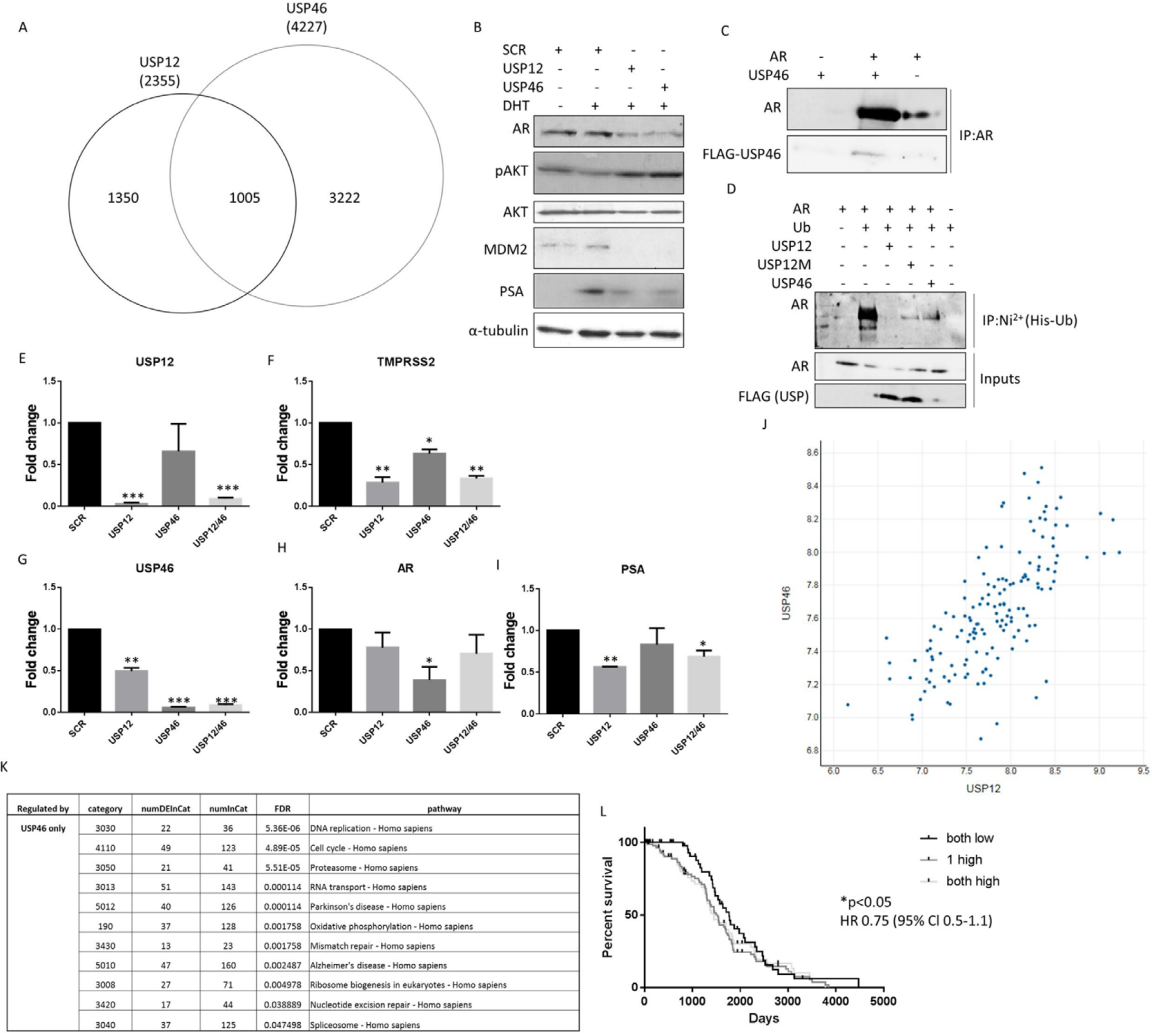
USP12 and USP46 share a significant functional overlap (**A**) Venn diagram of genes significantly affected (FDR <0.05) by 96 h of USP12 or USP46 silencing in LNCaP cells as analysed by RNA sequencing of three separate biological experiments. (**B**) Western analysis of LNCaP cell lysates following treatment with siRNA for 96 h, as indicated. (**C**) USP46 and AR interaction determined by immunoprecipitation following overexpression. (**D**) Deubiquitinase activity of wild type USP12 and its catalytically deficient mutant form (C48A USP12M [37]), and USP46 in COS-7 cells transfected with plasmids for 96 h as indicated. Cells were treated with MG-132 for the final 16h followed by denaturing immunoprecipitation. (**E**–**I**) Gene transcription in LNCaP cells following treatment with USP12 and USP46 siRNA for 96 h. Cells were grown in SDM for 72 h followed by 24 h in 10 nM DHT. (**J**) Correlation between USP12 and USP46 transcript levels in PC patient samples [41]. (**K**) KEGG pathway analysis of genes regulated exclusively by USP46 in transcriptomic analysis. (**L**) Relapse-free survival of PC patients based on their USP12 and USP46 levels (*n* = 142) [41].

To validate the shared role of USP12 and USP46 in prostate cancer cell biology we individually silenced both genes in PC cells and performed full transcriptome analysis to establish the extent of their shared functions. Over 40% of genes targeted by USP12 (1005/2355) were commonly regulated by USP46. USP46 shared a significant but smaller proportion of its targets with USP12 (24%; 1005/4227) (Figure 2A) indicating a more distinct function. We found a small group of genes (183) that were only affected when both USP12 and USP46 were silenced together, this group most likely represents genes were high level of redundancy in the function of these enzymes requires both to be silenced for any effect to be observed. We also found that silencing either USP12 or USP46 had the same effect on reducing AR, MDM2 and PSA protein levels whilst silencing either DUB activated the AKT signalling (pAKT) (Figure 2B). Following the overexpression of USP46 and AR proteins in COS-7 cells, immunoprecipitation of AR was shown to co-precipitate USP46 demonstrating interaction between the two proteins (Figure 2C). Additionally co-expression of USP46 stabilised the AR protein levels as seen in the input samples (Figure 2D) similarly to what we observe for USP12 [37]. Furthermore, overexpressed USP46 was able to deubiquitinate AR (Figure 2D), similarly to our previous observations for USP12 [37]. Consequently, USP46 silencing significantly affected transcript levels of multiple AR target genes, including *KLK3 (PSA), KLK2, NDRG1* and *UBE2C*, as demonstrated through RNA sequencing. Similarly, in our qPCR analysis, *PSA* and *TMPRSS2* levels were decreased upon both USP12 and USP46 silencing confirming their overlapping role in AR biology (Figure 2E–2I). We performed an *in silico* analysis of prostate cancer patient samples and demonstrated a significant correlation (0.7) between USP12 and USP46 expression (http://cancergenome.nih.gov/) [41] (Figure 2J). This data is consistent with the properties of these two highly homologous proteins, interacting with common protein partners, UAF1 and WDR20 [14, 15, 18, 40] (Supplementary Figure 1). Interestingly, both USP12 and USP46 affect the P53 pathway including transcript levels of *MDM2*, *BAX*, *NOXA* and *FOXO-3* and AR signalling including expression of *KLK3*, *KLK2* and *NDRG1* target genes [42, 43]. Consistent with a large number of genes being exclusively regulated by USP46 this DUB was observed to control multiple signalling pathways independently of USP12 (Figure 2K and Supplementary Figures 2–6). Functional overlap between USP12 and USP46 was confirmed in clinical samples, increase in just one of these enzymes was predictive of shortened relapse-free survival while increase of both transcript levels didn't convey any further predictive value (Figure 2L).

### Galeterone inhibits USP12 and USP46 in prostate cancer cells

**Figure 3:**
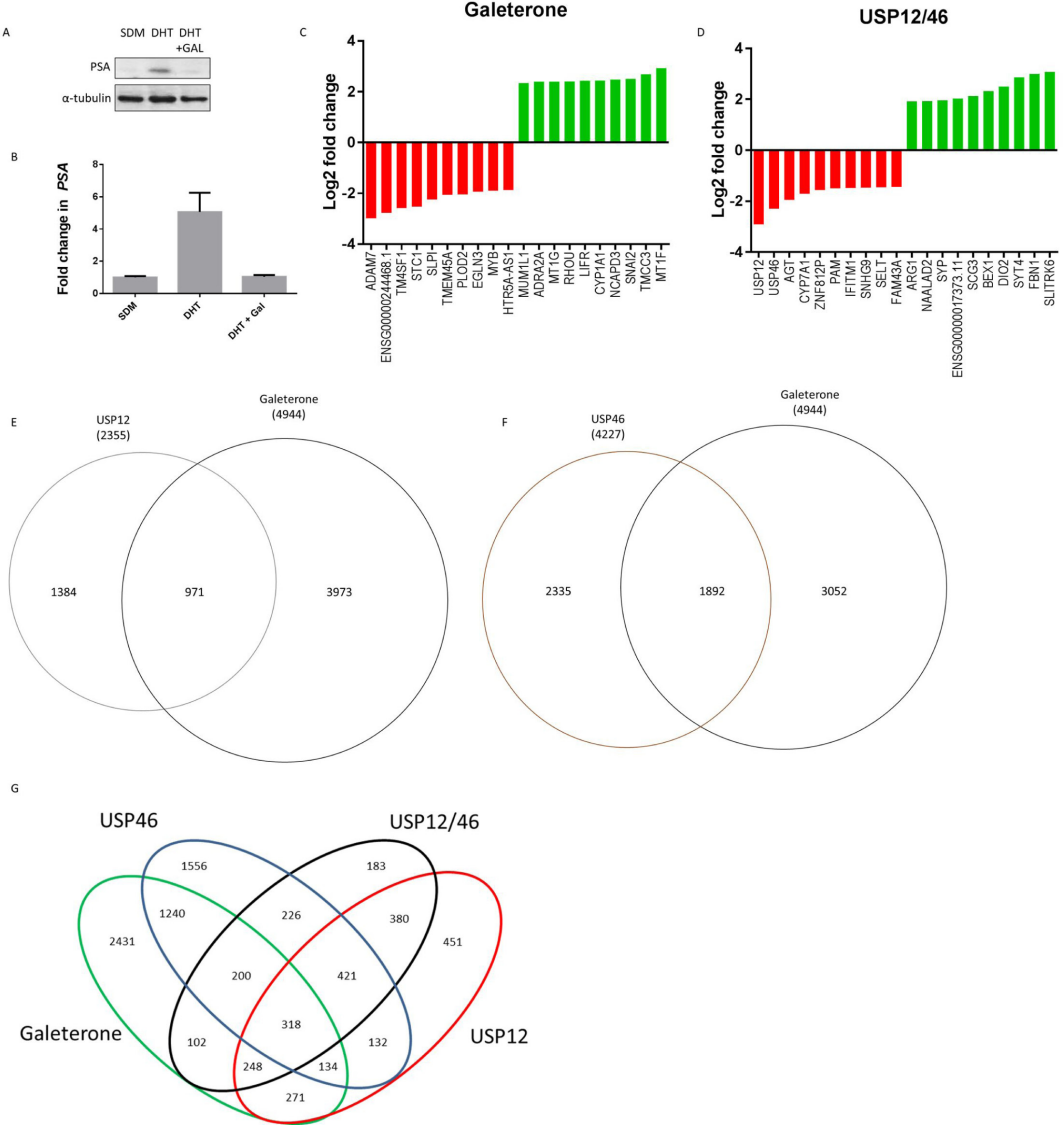
Galeterone targets USP12 and USP46 in PC cells (**A**–**B**) Galeterone downregulates PSA mRNA and protein expression. LNCaP cells grown in SDM for 72 h followed by 24 h in 10 nM DHT. Cells were treated with galeterone at 9.4 uM for 72 h followed by western blotting (A) and qPCR (B) analysis. (**C**–**D**) Genes most affected by 72 h galeterone treatment (C) or siUSP12/USP46 (D) as analysed by RNA sequencing. (**E**–**F**) Venn diagrams of genes affected (FDR <0.05) by galeterone treatment compared to USP12 silencing (E) or USP46 silencing (F), as analysed by RNA sequencing. (**G**) Venn diagram showing genes affected by one or more conditions (galeterone treatment, siUSP12, siUSP46 or siUSP12/USP46), determined by RNA sequencing.

**Figure 4:**
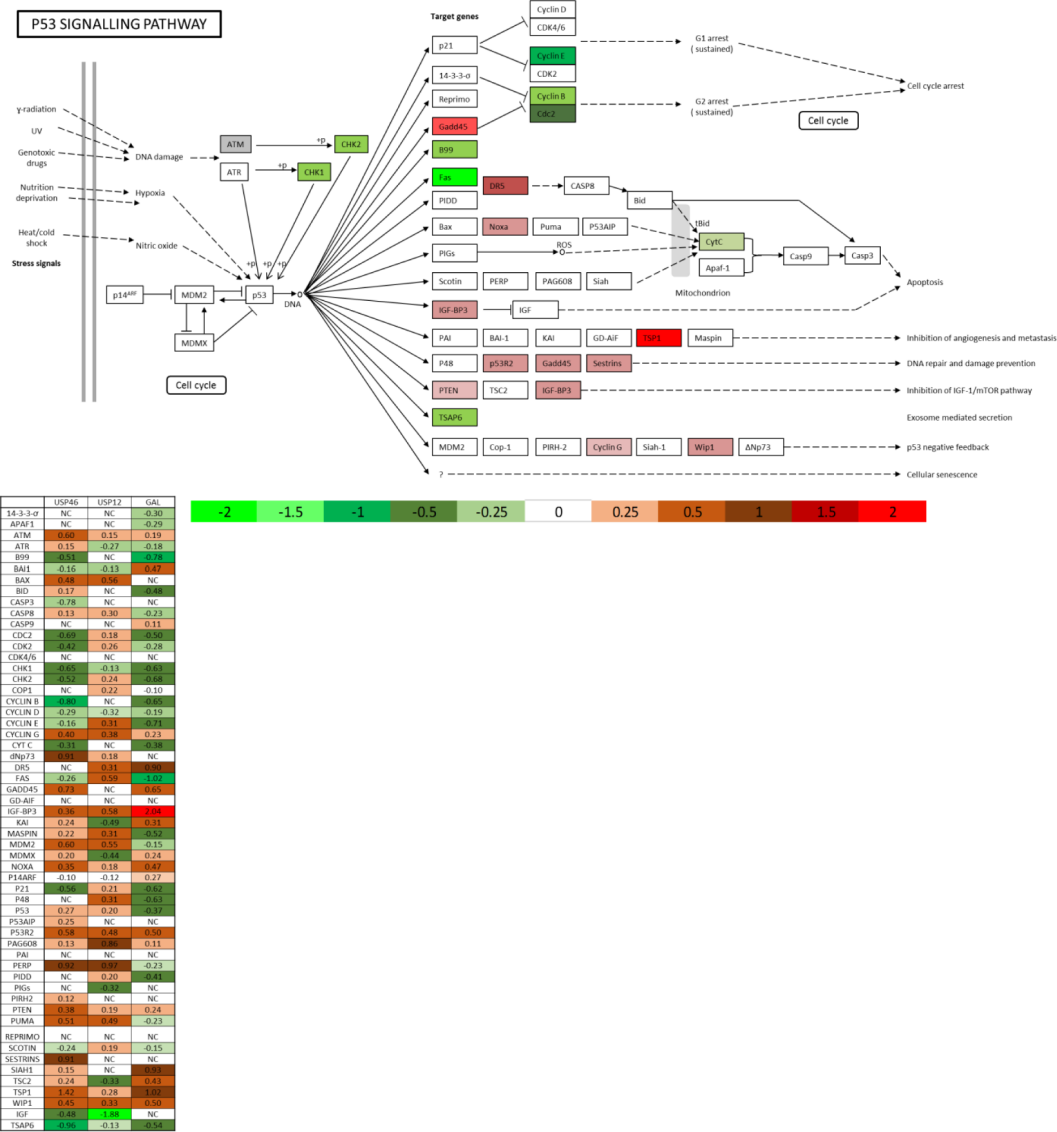
USP12, USP46 and galeterone all control the P53 pathway P53 KEGG pathway analysis comprised of genes regulated commonly by galeterone and at least one of the USP12/USP46 DUBs in the RNA sequencing experiments.

Galeterone is a novel antiandrogen that inhibits AR target gene expression, including PSA (Figure 3A–3B). We analysed the global effects of galeterone treatment at GI_50_ 9.4 μM on LNCaP PC cells by RNA sequencing. To investigate if galeterone controls AR and MDM2 protein stability via USP12 and USP46 inhibition we compared the PC transcriptome after galeterone treatment to siUSP12, siUSP46 and siUSP12/46 transcriptome. Galeterone treatment significantly affected transcript levels of 4944 genes compared to 2078 genes significantly affected by combined USP12 and USP46 silencing. The top 10 and bottom 10 most affected transcripts following galeterone treatment (Figure 3C) and joint USP12/46 gene silencing (Figure 3D) are shown. Over 40% of all the genes affected by either USP12 silencing (Figure 3E) or USP46 silencing (Figure 3F) were also similarly affected following galeterone treatment. Approximately 50% (2431/4944) of the genes affected by galeterone were not overlapped with any of the other treatment arms (siUSP12, siUSP46 or siUSP12/46) (Figure 3G), highlighting the wider cellular consequences of galeterone treatment as its targets include CYP17 lyase inhibition, AR antagonism, and induction of AR degradation as well as USP12 and USP46 inhibition. We performed KEGG pathway analysis of the 2431 genes exclusively regulated by galeterone and not USP12 or USP46 and determined that ‘ribosome’ was the only pathway significantly comprised within this gene list (FDR 4.2e-27) (Supplementary Figure 7). However, multiple pathways were deregulated by galeterone treatment in the same way as by USP12 and USP46 silencing. Galeterone, siUSP12 and siUSP46 all resulted in the upregulation of the crucial cancer associated pathway; P53 signalling (Figure 4). Additionally galeterone and siUSP46 had the same effect on multiple pathways involved in carcinogenesis and therapy resistance including cell cycle, ribosome biogenesis and RNA transport, spliceosome and DNA regulation at multiple levels with pyrimidine metabolism, DNA replication, nucleotide excision repair, mismatch repair and homologous recombination (Supplementary Figure 8–16). This confirms our biochemical data and demonstrates that galeterone inhibits USP12 and USP46 in PC cells.

To investigate whether the overlap between galeterone and USP12/46 target genes is a consequence of DUB enzymatic inhibition, we investigated USP complex transcript levels upon galeterone treatment. Galeterone treatment did not significantly affect *USP12* (adj. *p* = 0.5) while it had a slight but significant effect on *USP46* transcript levels (log2Fold = 0.3; adj. *p* = 0.003). Similarly galeterone inhibition did not have a significant effect on transcript levels of USP12 and USP46 complex members *UAF1* (adj. *p* = 0.4) and *WDR20* (adj. *p* = 0.25). This demonstrates that galeterone likely inhibits USP12 and USP46 enzymatic complexes at the protein level by inhibiting their deubiquitinating activity.

### Galeterone affects AR half-life and stability via USP12 and USP46 inhibition

**Figure 5:**
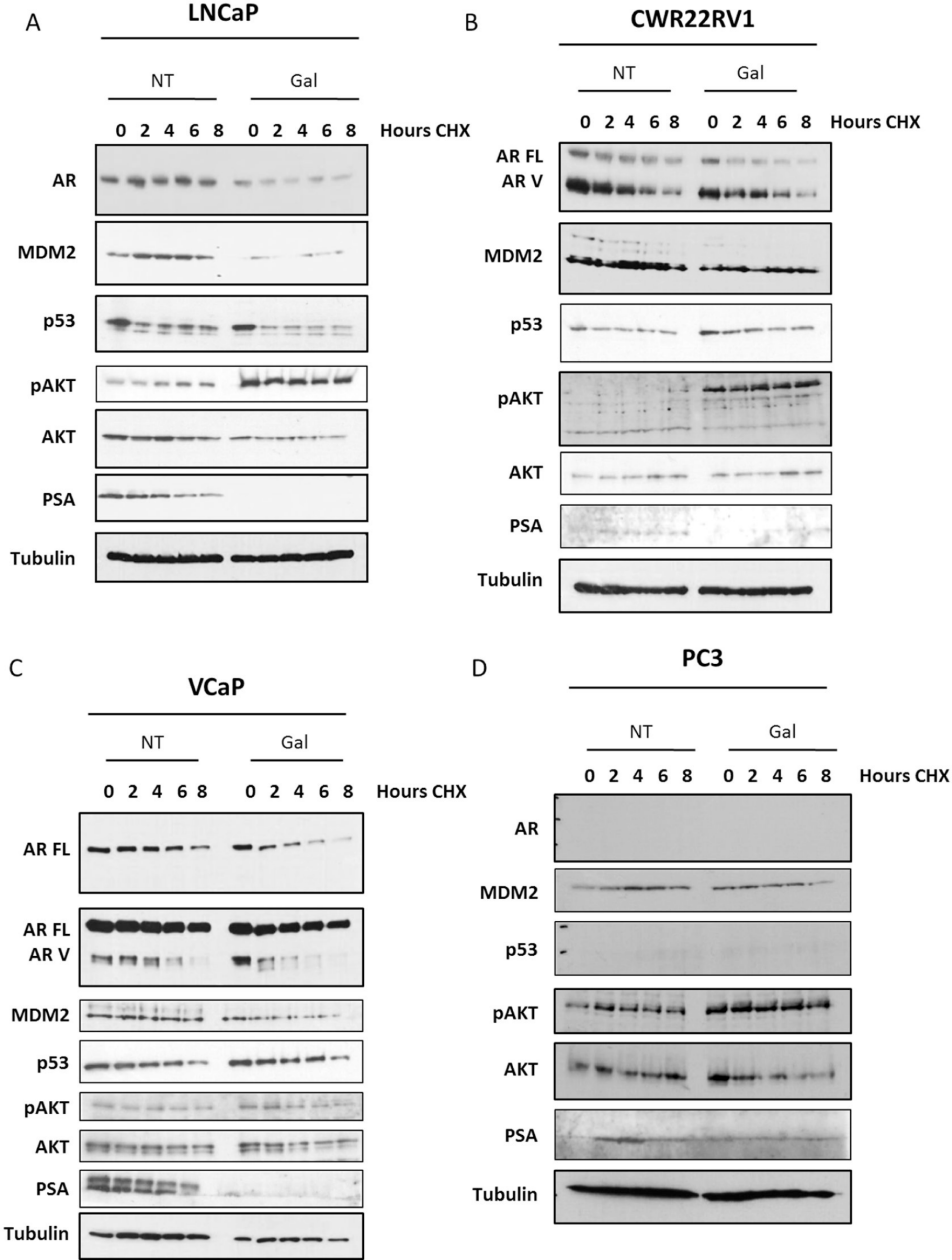
Galeterone controls AR FL and MDM2 protein stability and AKT phosphorylation (**A**–**D**) Cell lines were seeded and following 24 h incubation treated with galeterone at 9.4 μM for 72 h. After the experiment was terminated cells were lysed and protein levels were analysed by Western blotting. In 5D the line represents the end of the gel.

We have demonstrated the capacity of USP12 and USP46 to deubiquitinate the AR (Figure 2D) [37] and showed their similar effect on MDM2 protein levels (Figure 2B) [65]. USP12 and USP46 also control AKT phosphorylation levels (Figure 2B) by deubiquitinating PHLPP and PHLPPL AKT phosphatases [20, 21]. As galeterone inhibits USP12 and USP46 we assessed its effects on each of these proteins. In AR positive, castration sensitive LNCaP cells galeterone treatment decreased AR levels and AR half-life, abolished AR target gene, PSA, protein levels and decreased the levels of MDM2 protein while stimulating AKT phosphorylation (Figure 5A). Similar effective reduction of full-length AR (AR FL) and MDM2 was observed in the AR FL and AR variant (AR V) positive CWR22Rv1 castrate resistant cells (Figure 5B) with a more pronounced effect on activation of AKT (pAKT) than in the LNCaP cell line (Figure 5A). In the AR amplified VCaP cell line (Figure 5C) and AR negative, PSA negative PC3 cells (Figure 5D) similar effects were observed. However, no significant effect of galeterone was observed on constitutively active shorter AR variants (Figure 5B–5C).

### Galeterone effectively inhibits cell growth in AR-dependent and AR-independent PC cell lines

**Figure 6:**
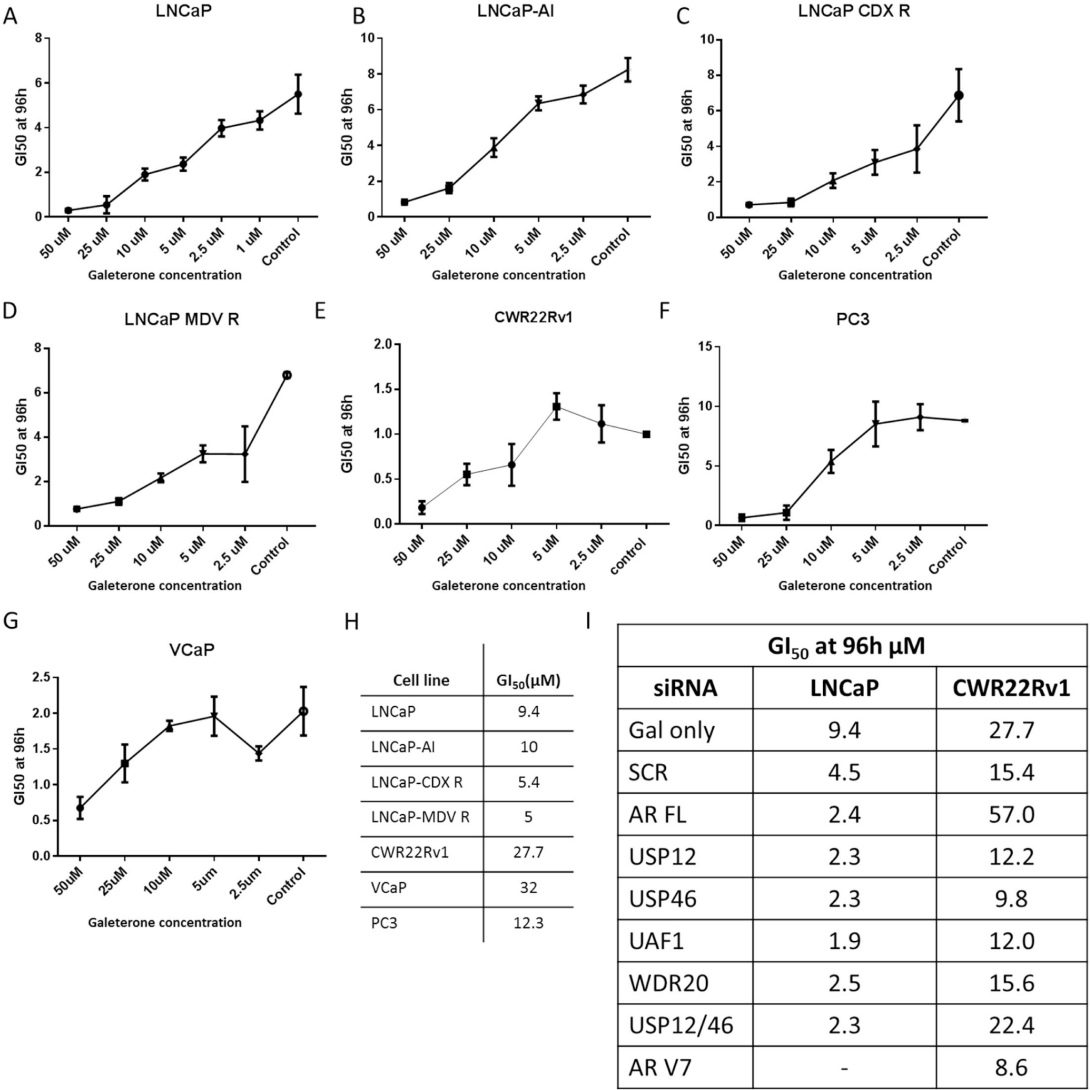
Galeterone inhibits PC cell growth (**A**–**G**) Cells were treated with galeterone for 96 h with treatment starting 24 h after seeding. GI50 was assessed using IncuCyte by live cell imaging every 6 hours. (**H**) Comparison of cellular GI50s, assessed by IncuCyte. (**I**) Comparison of galeterone GI50 values in cells treated with galeterone and siRNAs. Cells were seeded and treated with siRNAs as indicated. Following 24 hours incubation cells were treated with varying concentrations of galeterone and GI50s were assessed using IncuCyte. All data represents the mean of at least three independent experiments ± SEM.

Therapy resistance is the main cause of prostate cancer mortality. To investigate whether galeterone could offer a therapeutic advantage in patients who develop resistance to currently available treatments we determined the growth inhibitory effects of galeterone in multiple cell lines representing different stages of PC progression. Galeterone was highly effective in inhibiting castration-sensitive LNCaP cell growth, a representative of early stage disease (Figure 6A). Galaterone was equally effective in inhibiting LNCaP cell growth when cells were cultured in the presence of androgens or in castrate conditions (Supplementary Figure 17). Additionally, GI_50_ values were comparable between LNCaP androgen sensitive cells and LNCaP-AI androgen independent cells (Figure 6B) as well as LNCaP cells which had acquired resistance to second line anti-androgens currently used in the clinic, Casodex (Figure 6C) and MDV-3100 (Figure 6D). For the AR negative PC3 prostate cancer cell line, established from an aggressive bone metastasis, galeterone was similarly effective in inhibiting cell growth when compared to LNCaP cells (Figure 6F). However, galeterone was substantially less effective in inhibiting growth of CWR22Rv1 and VCaP cells that harbour constitutively active AR variants (Figure 6G–6HG). These experiments were extended to determine the effects of galeterone when combined with silencing multiple members of the USP-AR interacting network. In the LNCaP cell line, silencing AR, USP12, USP46 or their cofactors UAF1 and WDR20 was found to enhance the inhibitory effects of galeterone by an average 2-fold when compared to SCR control (Figure 6I). Conversely, in the CWR22Rv1 cell line silencing AR FL alone decreased galeterone efficiency by 4 fold compared to SCR control and silencing USP12 or USP12-USP46 complex members had no significant effect on galeterone efficacy. Interestingly, improvement in galeterone GI_50_ efficacy was observed when combined with USP46 or AR V7 silencing suggesting that AR V7 expression conveys resistance to galeterone (Figure 6I).

### USP12 is unable to bind or impact on the protein stability of AR Vs lacking the ligand binding domain

**Figure 7:**
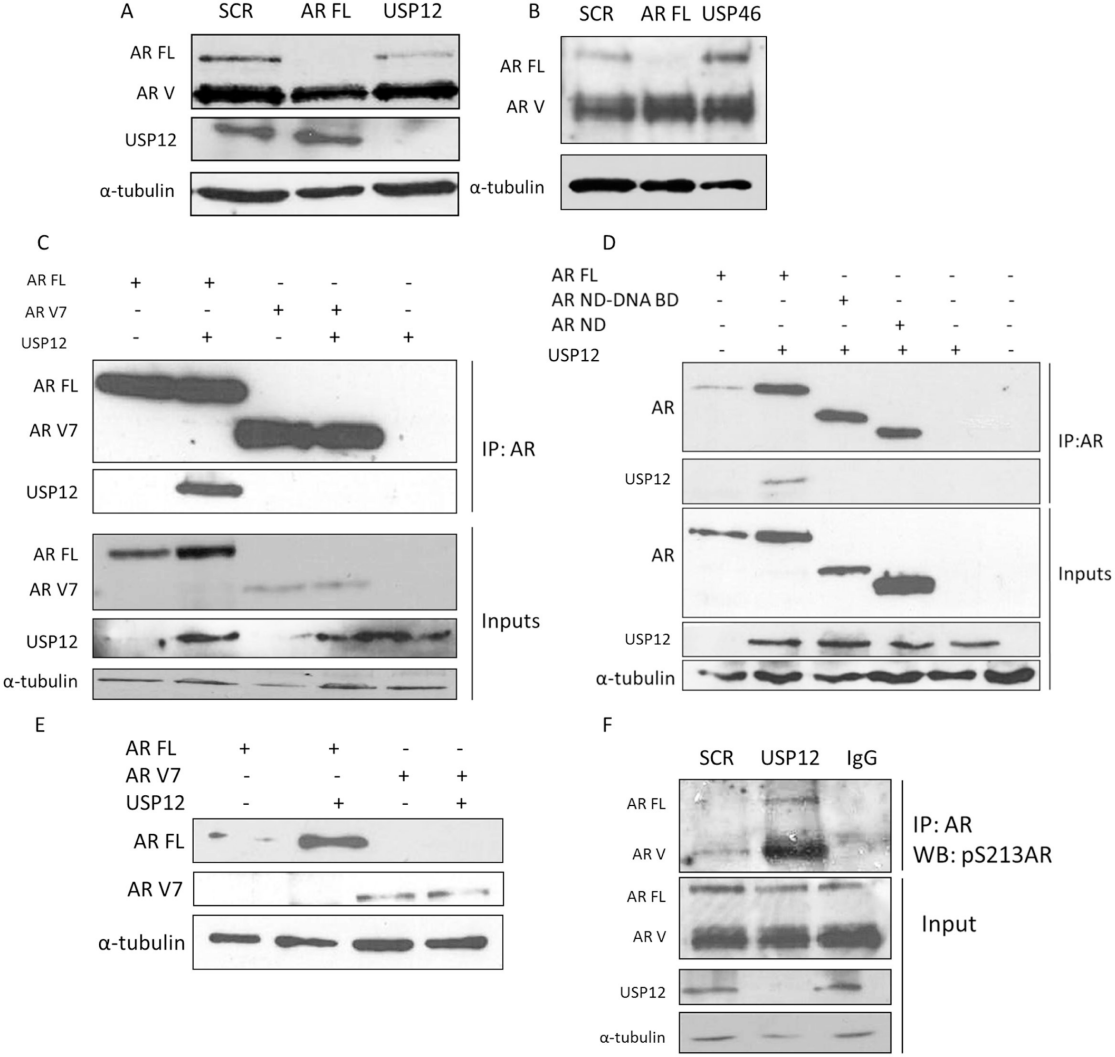
USP12 does not interact with AR-V (**A**–**B**) CWR22Rv1 cells treated with siRNA for 96 h and protein expression evaluated by Western blotting. (**C**–**D**) Plasmids were overexpressed in COS-7 cells for 72 h followed by immunoprecipitation and Western blotting for the indicated proteins. (**E**) Plasmids were overexpressed in COS-7 cells for 72 h as indicated and Western blotting performed for AR FL and AR V7 proteins. (**F**) CWR22Rv1 cells were treated with siRNA for 96 h followed by immunoprecipitation and Western blotting.

To investigate whether the diminished galeterone efficacy in CWR22Rv1 cells was a consequence of inability of USP12 activity to be directed towards AR Vs we silenced USP12 in CWR22Rv1 cells. Following depletion of USP12, the level of AR FL protein was reduced but we failed to observe any effect of USP12 or USP46 depletion on AR V protein expression (Figure 7A–7B). We reasoned that the failure for USP12 and USP46 to impact on AR V levels may be due to an inability for USP and AR Vs to physically interact. We overexpressed USP12 alongside AR FL or clinically relevant AR V7 in COS-7 cells and immunoprecipitated AR constructs. Although we were able to confirm the USP12- AR FL interaction we could not detect any interaction between USP12 and AR V7 (Figure 7D). To validate this result we overexpressed USP12 with AR FL, N-terminus of AR (AR ND) and AR N-terminus with DNA binding domain (AR ND-DNA BD) to localise the USP12-AR interaction. We found that USP12 was unable to interact with both AR constructs lacking the C-terminal ligand binding domain (Figure 7C). We speculated that this might be because, as we recently reported, the AR requires its C-terminus for ubiquitination [42] and therefore may require this domain for recognition by deubiquitinating enzymes. The requirement of the AR ligand binding domain to ensure an interaction between USP12 and AR is supported by our results in Figure 7D showing that AR FL is stabilised following USP12 overexpression whereas the AR V7 proteins levels are unaffected (Figure 7E). However, USP12 was still able to exert an indirect effect on AR Vs by controlling its phosphorylation through AKT activation (Figure 7F), confirming that AR Vs are still phosphorylated at S213 while this phosphorylation is most likely to be of no consequence for AR V activity as phosphorylation at this site reportedly decreases AR-androgen binding and targets AR for ubiquitination by MDM2 [44], both processes that seem irrelevant to AR V biology.

### USP12 inhibition in AR V positive lines drives the AR V expression profile

**Figure 8:**
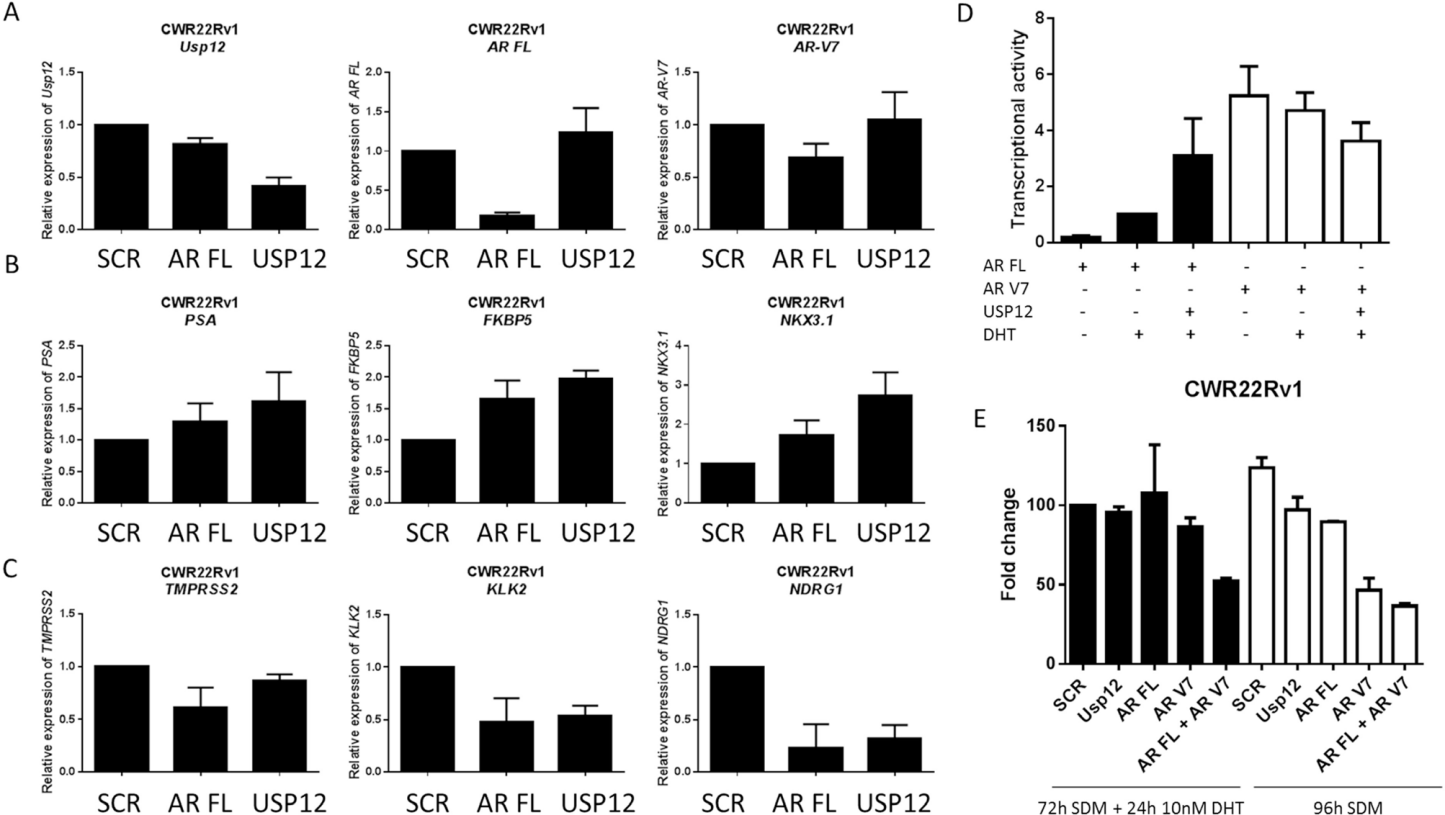
USP12 silencing promotes an AR V7 expression profile (**A**–**C**) Transcript levels following treatment with USP12 siRNA for 96 h in CWR22Rv1 grown in steroid containing media. Data are a mean of three independent experiments normalised to SCR. (**D**) Transcriptional activity using luciferase in HEK293T cells transfected with the indicated plasmids and pARE3-luc [37] for 72 h in steroid depleted conditions followed by addition of 10 nM DHT for 24 h where indicated. Data are a mean ± SEM of three independent experiments. (**E**) Cellular proliferation of CWR22Rv1 after 96 h in SDM ± 10 nM DHT following siRNA depletion of the indicated transcripts. Cellular proliferation was assessed by cell counting and data presented is the mean ± SEM of three independent experiments.

Our data suggests that USP12 is unable to modulate AR V levels or activity but is capable of targeting AR FL. To confirm the lack of USP12 activity towards AR Vs we analysed transcript levels of genes preferentially targeted by either AR FL or AR V7 following AR FL and USP12 silencing in the CWR22v1 cell line (Figure 8A). We observed that the transcript levels of *PSA*, *FKBP5* and *NKX3-1* (Figure 8B) increase whereas *TMPRSS2*, *KLK2* and *NDRG1* (Figure 8C) transcript levels decreased following USP12 silencing. A similar pattern of change in transcript levels for these genes was observed when AR FL was silenced (Figure 8B, 8C). This is in agreement with published literature where increased V7 was reported to regulate *FKBP5* and *NKX3-1* but not *TMPRSS2, KLK2* or *NDRG1* [45]. Our previous observations for the LNCaP cell line that express AR FL but not AR Vs have demonstrated that USP12 silencing causes a decrease in transcript expression of all the AR target genes [37] supporting our data that AR Vs are not targeted by USP12 in the AR V expressing CWR22Rv1 cell line. Additionally, in an androgen responsive luciferase expression system, we found USP12 over-expression to efficiently co-activate AR FL transcriptional activity following DHT stimulation however, USP12 over-expression had no effect on AR V7 transcriptional activity (Figure 8D). Following USP12 silencing in CWR22Rv1 cells we observed only a marginal effect on proliferation in either androgen-deprived or androgen-replete conditions (Figure 8E). Silencing of either AR FL and/or AR V7 in the presence or absence of androgen confirmed the importance of AR V7 in maintaining CWR22Rv1 proliferative ability in castrate conditions (Figure 8E). As expected, the silencing of USP12 or AR FL in the LNCaP cell line had similar effects on LNCaP proliferation (Figure 8E).

## DISCUSSION

We are first to report USP46 to be a deubiquitinating enzyme for AR. USP46 is highly homologous to USP12 in sequence, NLS and UAF1 and WDR20 co-activator requirement [13, 14], consequently it is unsurprising that they would both target AR for deubiquitination. These two enzymes, even though very homologous with each other, share limited similarity with other USPs [46, 47]. In this manuscript we have performed global transcriptomic analysis of USP12 and USP46 regulated pathways in PC cells. We have uncovered that, in agreement with our biochemical data, both of these enzymes control AR signalling and the P53 pathway, additionally USP12 generally overlapped in function with USP46. USP46 however, affects more cellular transcripts than USP12 and consequently it has a number of cellular pathways that it controls exclusively of USP12 including spliceosome and proteasome assembly and multiple DNA pathways. To our knowledge this is the first global analysis investigating the cellular roles of USP12 and USP46 in PC.

In this manuscript we focused on uncovering the molecular mechanism of galeterone's activity. It has been previously reported that galeterone controls AR and MDM2 protein stability while the mechanism was not determined [8, 9]. We screened a large panel of deubiquitinating enzymes and discovered that galeterone specifically inhibited USP12 and USP46, while it had no effect on any other DUBs. Further analysis showed a significant functional overlap between galeterone treatment and USP12/USP46 silencing. Through inhibiting USP12 and USP46 enzymatic activity galeterone was able to inhibit cell growth even in AR negative PC models, highlighting the role of USP12 and USP46 in P53 and AKT pathways regulation [20, 21, 63, 64]. We propose a model in which galeterone controls AR through CYP17 inhibition and consequences of its direct binding to AR, whereas USP12 and USP46 inhibition controls P53 and AKT pathways (Supplementary Figure 18). This manuscript provides a proof of principle for USP12 and USP46 targeted inhibition in cancer therapy. Effective treatment efficacy in AR negative PC3 line indicates that this strategy may be valuable not only in prostate cancer. Recently, studies into USP1 inhibition have shown high efficacy in lung cancers including reversing cisplatin resistance [48–50], GBM [51], leukaemia [52] and osteosarcoma [53]. USP1, similarly to USP12 and USP46, deubiquitinates AKT phosphatases PHLPP and PHLPPL [54] and relies on UAF1 for its enzymatic activity, while it does not bind to WDR20 or any other additional proteins [15]. This suggests that USP12/46 inhibition could be explored in a similar setting. With the recent structural discovery of USP12-UAF1 [16, 18] and USP46-UAF1 [19], the development of highly specific inhibitors targeting the USP-UAF1 interaction will be possible.

We have demonstrated galeterone to be highly effective in androgen sensitive, androgen independent, anti-androgen resistant and AR negative PC models. However, we observed that galeterone was substantially less effective in inhibiting constitutively active AR Vs. This might be explained by the fact that USP12 and USP46 also have no effect on AR Vs, potentially because shorter AR variants are not ubiquitinated [42]. Consequently, even though USP12 and USP46 can still control AR V S213 phosphorylation levels, by controlling the AKT activity, this phosphorylation site has no effect on AR V stability or activity. This might explain recent results of the phase 3 clinical trial where galeterone was compared directly to enzalutamide in men with metastatic CRPC and AR-V7 expression. Our data are in conflict with observations made by Kwegyir-Afful [8] and Duo who reported that galeterone treatment resulted in substantial reductions in AR V levels in CWR22Rv1 cells. However, a similar lack of galeterone's activity against AR V has been previously reported [9]. Our data indicates that inhibition of USP12 and USP46 activity with galeterone, or other compounds may be a very effective anti-tumour strategy, however galeterone effects in those PC patients expressing AR V may be limited.

## MATERIALS AND METHODS

### Antibodies and plasmids

anti-AR (Santa Cruz Biotechnology; N20 clone), anti-H2A.Z [55], anti-MDM2 (Santa Cruz N20 and SMP14), anti-FLAG, anti-USP12 and anti-α-tubulin (Sigma), anti-PSA and anti-P53 (Dako), and anti-ubiquitin and anti-pAKT (Santa Cruz Biotechnology) antibodies were included in this project. Plasmids used were pARE3-Luc, pCMV-β-gal [56], pFLAG-USP12 wild type and C48A mutant [37], pHA-Ubiquitin, p-FLAG-His-AR, pFLAG- AR-V7 and pDEST-IRIS-HA-FLAG-USP46 [14].

### Cell culture, transfections

LNCaP, PC3, CWR22Rv1, HEK293T and COS-7 cells (all purchased from the ATCC); LNCaP-Cas R, LNCaP-MDV R and LNCaP-AI are variant cell lines derived in-house by serial exposure to Bicalutamide, MDV3100 and steroid depleted conditions, respectively [57] were cultured in RPMI 1640 medium with 2 mM L-glutamine (Invitrogen) supplemented with 10% (v/v) foetal calf serum (FCS) or steroid depleted serum (SDM) at 37° C in 5% CO_2_. VCaP cells kindly donated by Professor Guido Jenster (Erasmus Medical Centre, Rotterdam) were cultured in DMEM medium with 2 mM L-glutamine (Invitrogen) supplemented with 10% (v/v) foetal calf serum (FCS) or steroid depleted serum (SDM) at 37° C in 5% CO_2_. Cell lines were never maintained for more than 30 passages or 3 months of continuous culturing. As per institutional policy, cell lines were tested for mycoplasma on a tri-monthly basis. Transfections were performed using TransIT-LT1 reagent (MirusBio) following the manufacturer's instructions. Luciferase assays were performed as previously described [37].

### Immunoprecipitation (IP)

Cells were seeded at 10^6^ cells/90-mm dish, transfected with 1 μg of plasmid when indicated, incubated for 72 h, and lysed directly into lysis buffer (50 mM Tris, pH 7.5, 150 mM NaCl, 0.2 mM Na_3_VO_4_, 1% Nonidet P-40, 1 mM PMSF, 1mM DTT, and 1 × protease inhibitors (Roche Applied Science)). Lysates were incubated with 1 μg of antibodies for 16 h at 4° C, and antibodies were pulled down using protein G-Sepharose beads (Invitrogen). For denaturing IPs, cells were subjected to 20 μM MG132 proteasomal inhibitor treatment for the final 16 h followed by collection into denaturing lysis buffer [37] prior to immunoprecipitation using Nickel beads.

### siRNA gene silencing and gene expression analysis

Cells were reverse transfected with siRNA using RNAiMax (Invitrogen) according to the manufacturer's instructions and incubated in culture medium for 96 h prior to cell lysis. siRNA sequences were as follows SCR: UUCUCCGAACGUGUCACGU[dT][dT]; *AR FL*: CCAUCUUUCUGAAUGUCCU[dTdT]; *USP12*: CAGAUCUCUUCCAUAGCAU[dTdT], *WDR20*: CGAG AAAGAUCACAAGCGA[dTdT] and *UAF1*: CAAAUU GGUUCUCAGUAGA[dTdT] [57]; *AR V7*: GUAGUUG UGAGUAUCAUGA[dT][dT] and *USP46*: GUCUCAAU GGUCUGGCUGU[dT][dT]. For RNA sequencing, RNA was extracted using the QIAGEN RNeasy Plus Mini Kit and all samples were sequenced from three separate biological experiments using Illumina's total stranded RNA prep kit with ribozero gold for library preparation with 100 bp paired end reads on the Illumina HiSeq 2500 platform, performed by AROS.

Reads were mapped to the reference human genome hg19 using STAR2-pass allowing up to two mismatches [58]. Per gene raw read counts for each sample were obtained using HTseq and Gencode version 19 [59]. Gene-level differential expression analysis was performed using DEseq2 [60]. *P* values were adjusted to control for the false discovery rate (FDR) using the Benjamini-Hochberg method. Differentially expressed genes from each comparison were tested for functionally enriched pathways and gene ontology terms using GOseq with a gene length bias correction on pathways annotated in KEGG database.

### Proliferation analysis

For proliferation analysis cells were seeded 24 hours prior to treatment with either DMSO (control) or galeterone for 96 hours. IncuCyte measurements of cellular occupation of the wells were taken every 6 hours. Cell growth rate was normalised to the time point zero and additionally in a separate set of experiments cell numbers were counted at 96 h to assess cellular proliferation [61].

### qPCR

For qPCR, RNA was extracted using Trizol (Invitrogen) according to manufacturer's instruction, and quantified using Nanodrop. Briefly, 1 μg of RNA was reverse transcribed with 1U of MMLV reverse transcriptase, 50 ng Oligo-dT and 630 μM dNTP (Promega). All qPCR was performed using the relative quantification method on three independent biological experiments and each sample was loaded in triplicate. qRT-PCR was conducted using SYBR^®^Green on 384-well optical reaction plates with the ABI 7900HT real-time PCR system. Results were normalised to HPRT1 expression. Primers were *HPRT1* F: 5′-GAACGTCTTGCTCGAGAGATGTG-3′, R: 5′-CCA GCAGGTCAGCAAAGAATTT-3′; *KLK2* F: 5′-AGCA TCGAACCAGAGGAGTTCT-3′, R: 5′-TGGAGGCTCA CACACTGAAGA-3′; *PSA* F: 5′-ACTGCATCAGGAAC AAAAGCGT-3′, R: 5′-TGTGGGAAGCTGTGGCTGA C-3′; *NDRG1* F: 5′-ACAACCCCCTCTTCAACTACG-3′, R: 5′-GCCAATAATGCTTTTCAGCCCA-3′; *AR F: 5*′-G CAAAGCCTAAAGCCAGAT-3′, R: 5′-GAGTTCATGG GTGGCAAAG-3′; *NKX3.1 F: 5*′-AGCCAGAAAGGCA CTTGGG-3′, R: 5′-GGCGCCTGAAGTGTTTTCA-3′; *TMPRSS2 F: 5*′-CTGCTGGATTTCCGGGTG-3′, R: 5′-T TCTGAGGTCTTCCCTTTCTCCT-3′; *FKBP5* F: 5′-GC AACAGTAGAAATCCACCTG-3′, R: 5′- CTCCAGAG CTTTGTCAATTCC-3′; *USP46* F: 5′- TCCGGGAGA ATGTGTTGGC -3′, R: 5′- GTGTGGCAATGCTGTGGA AAA -3′; *USP12* F: 5′-CAGCCTTCCAGTCATTGGCA-3′, R: 5′-ATCAATACGGCACAGATTCCG-3′; *WDR20* F: 5′-TGCACCAGATCTAGAACTTGAAT-3′, R: 5′-TAT ACTCCCAGGAGGATGACTG-3′; *UAF1* F 5′-GCT GATTGGTATGGACCGA-3′, R: 5′-TCTGCTTCCCT GGGGACAG-3′; *AR V7* F: 5′-AACAGAAGTACCTG-3′, R: 5′-TCAGGGTCTGGTCATTTTTG-3′.

### Statistical analysis

All data was first tested for its Gaussian distribution. Normally distributed data was tested with *t*-test or ANOVA with Dunn's multiple comparisons where more groups where compared.

## SUPPLEMENTARY MATERIALS FIGURES AND TABLES


