# Supplementary material for: The novel anti-androgen candidate galeterone targets deubiquitinating enzymes, USP12 and USP46, to control prostate cancer growth and survival
Source: Oncotarget. 2018 May 18;9(38):24992–5007. doi: 10.18632/oncotarget.25167 (PMC5982776; doi:10.18632/oncotarget.25167)
Supplement: Supplementary file 1 [file oncotarget-09-24992-s001.pdf]

The novel anti-androgen candidate galeterone targets deubiquitinating enzymes, USP12 and USP46, to control prostate cancer growth and survival

SUPPLEMENTARY MATERIALS

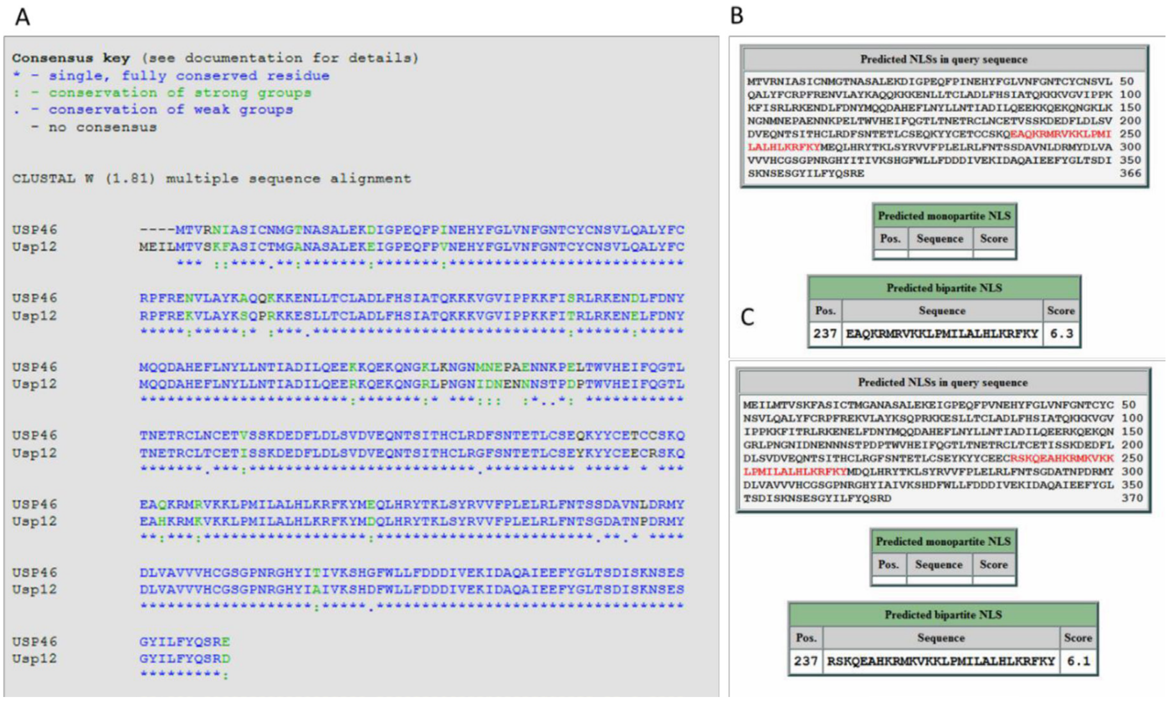

Supplementary Figure 1: USP12 and USP46 proteins are highly homologous. (**A**) ClustalW alignment of USP12 and USP46 (http://workbench.sdsc.edu). (**B–C**) cNLS mapper prediction of USP46 (B) and USP12 (C) bipartite nuclear localisation sequence [63].

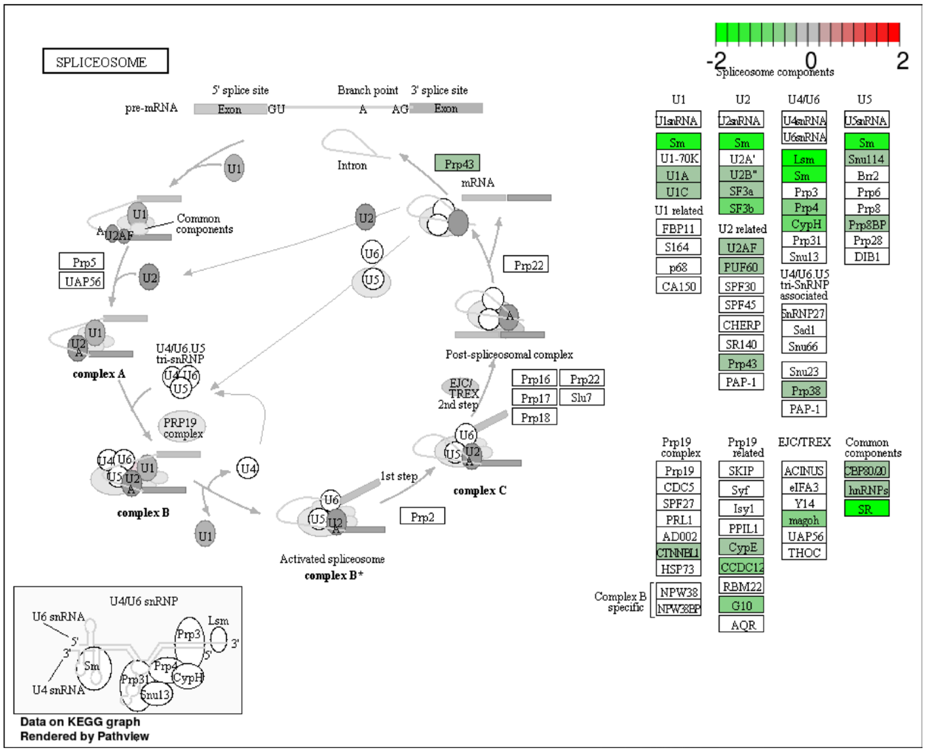

Supplementary Figure 2: USP46 controls spliceosome pathway. Spliceosome KEGG pathway analysis comprised of genes regulated exclusively by USP46 and not USP12.

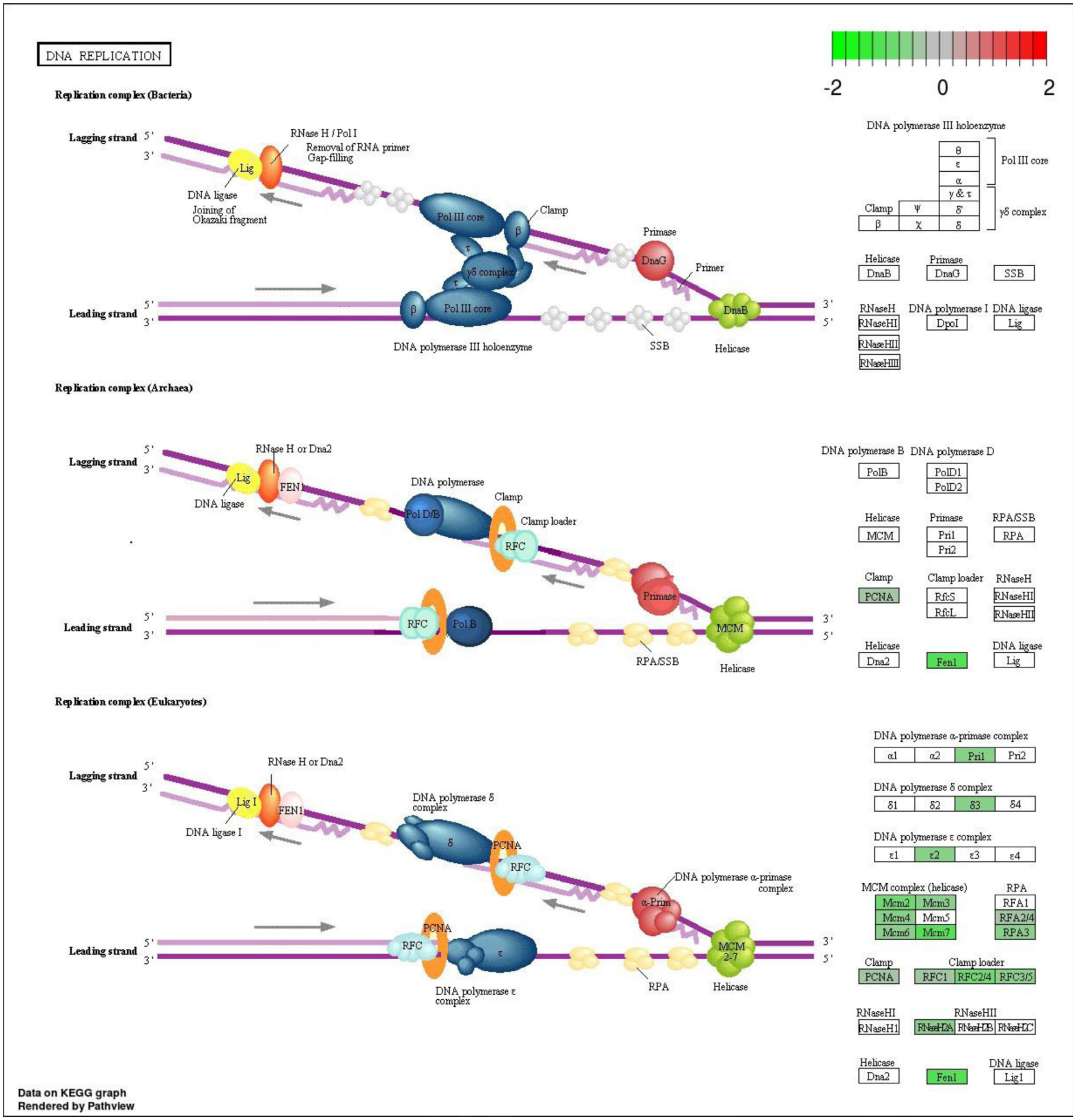

Supplementary Figure 3: USP46 controls DNA replication pathway. DNA replication KEGG pathway analysis comprised of genes regulated exclusively by USP46 and not USP12.

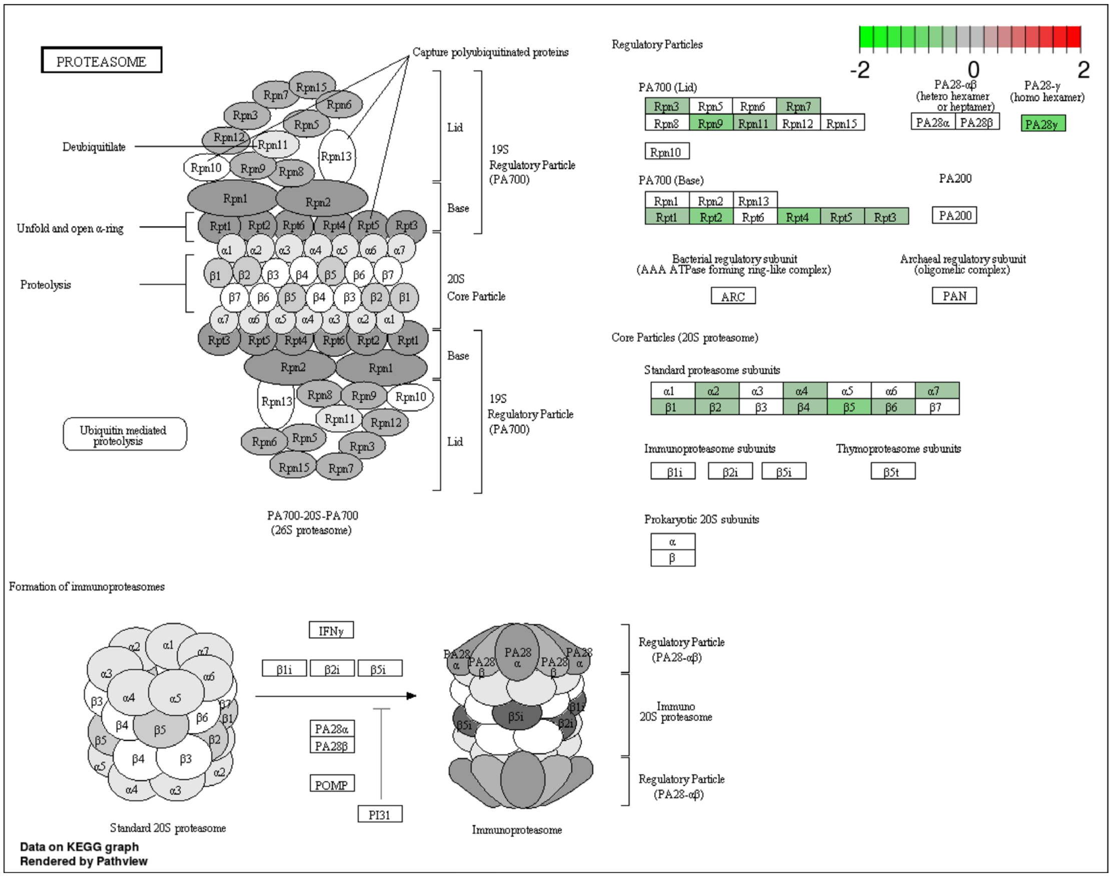

Supplementary Figure 4: USP46 controls proteasome pathway. Proteasome KEGG pathway analysis comprised of genes regulated exclusively by USP46 and not USP12.

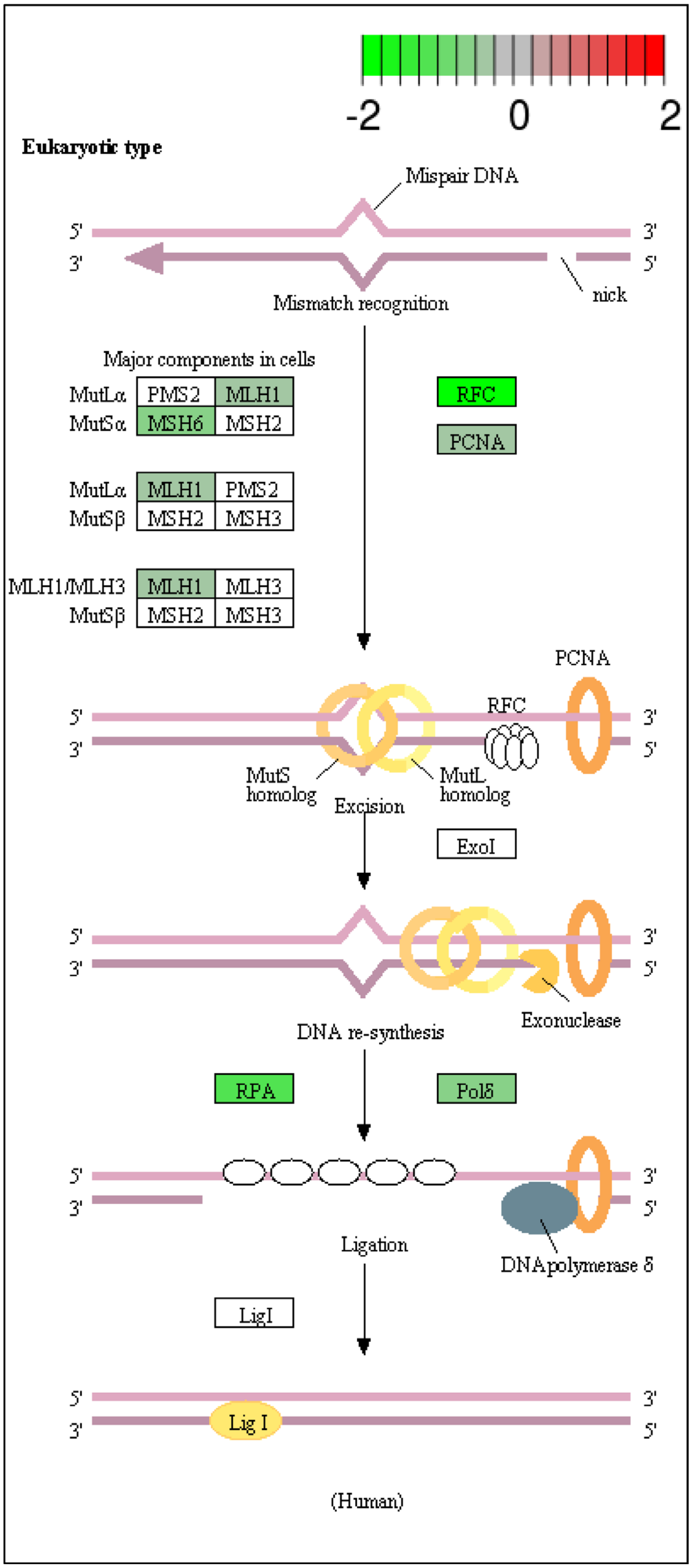

Supplementary Figure 5: USP46 controls mismatch-repair pathway. Mismatch-repair KEGG pathway analysis comprised of genes regulated exclusively by USP46 and not USP12.

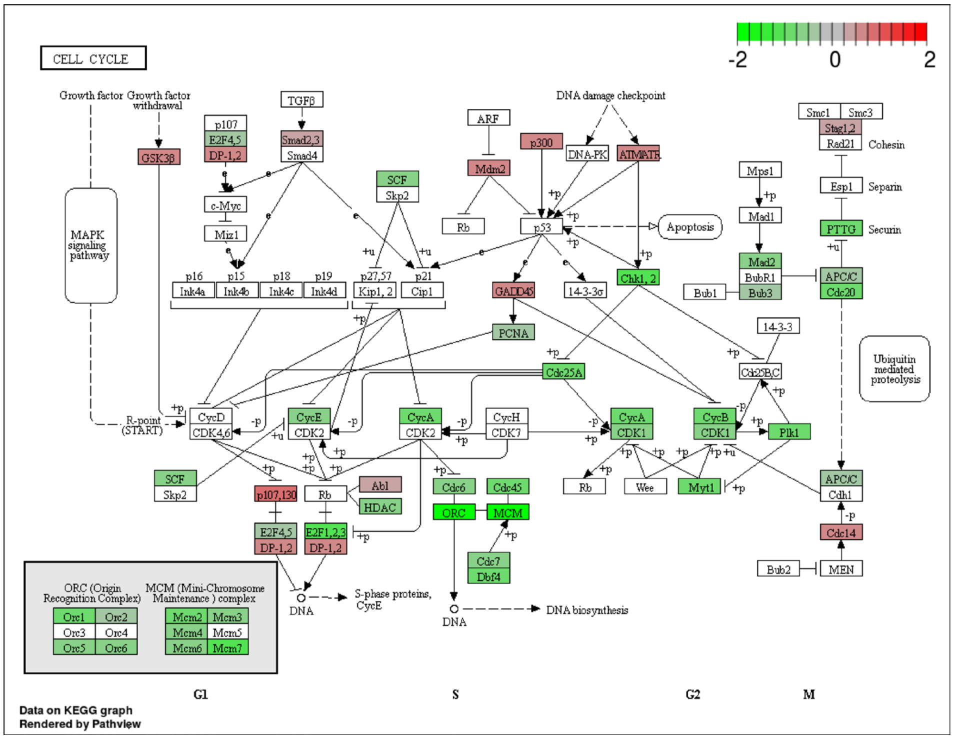

Supplementary Figure 6: USP46 controls cell cycle pathway. Cell cycle KEGG pathway analysis comprised of genes regulated exclusively by USP46 and not USP12

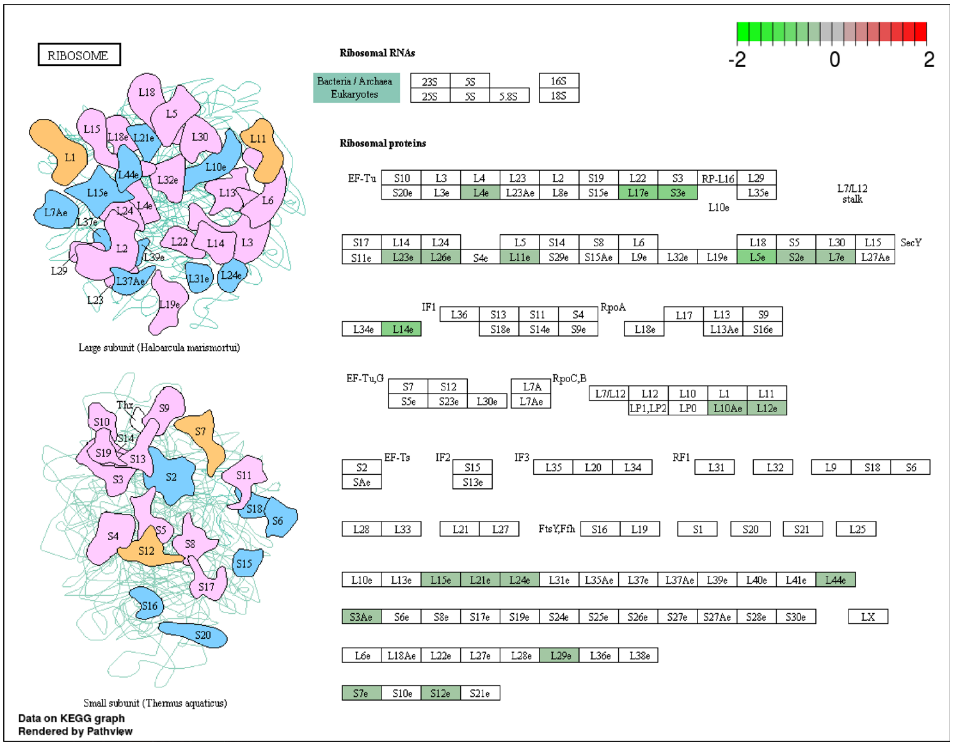

Supplementary Figure 7: Galeterone controls the ribosomal pathway. Ribosome KEGG pathway analysis comprised of genes regulated exclusively by galeterone and not USP12, USP46 or USP12/USP46.

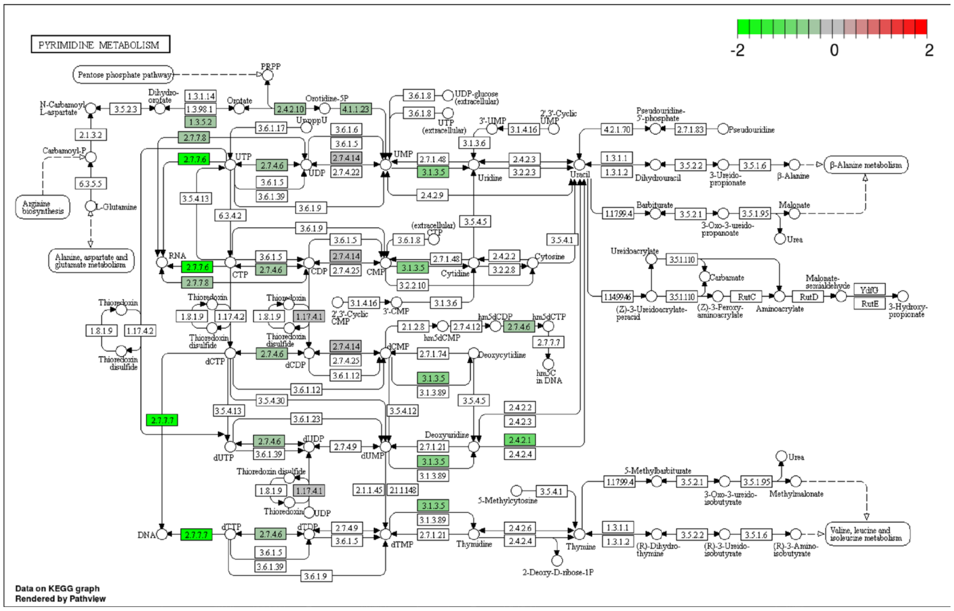

Supplementary Figure 8: USP46 and galeterone control pyrimidine metabolism pathway. Pyrimidine metabolism KEGG pathway analysis comprised of genes regulated commonly by USP46 and galeterone.

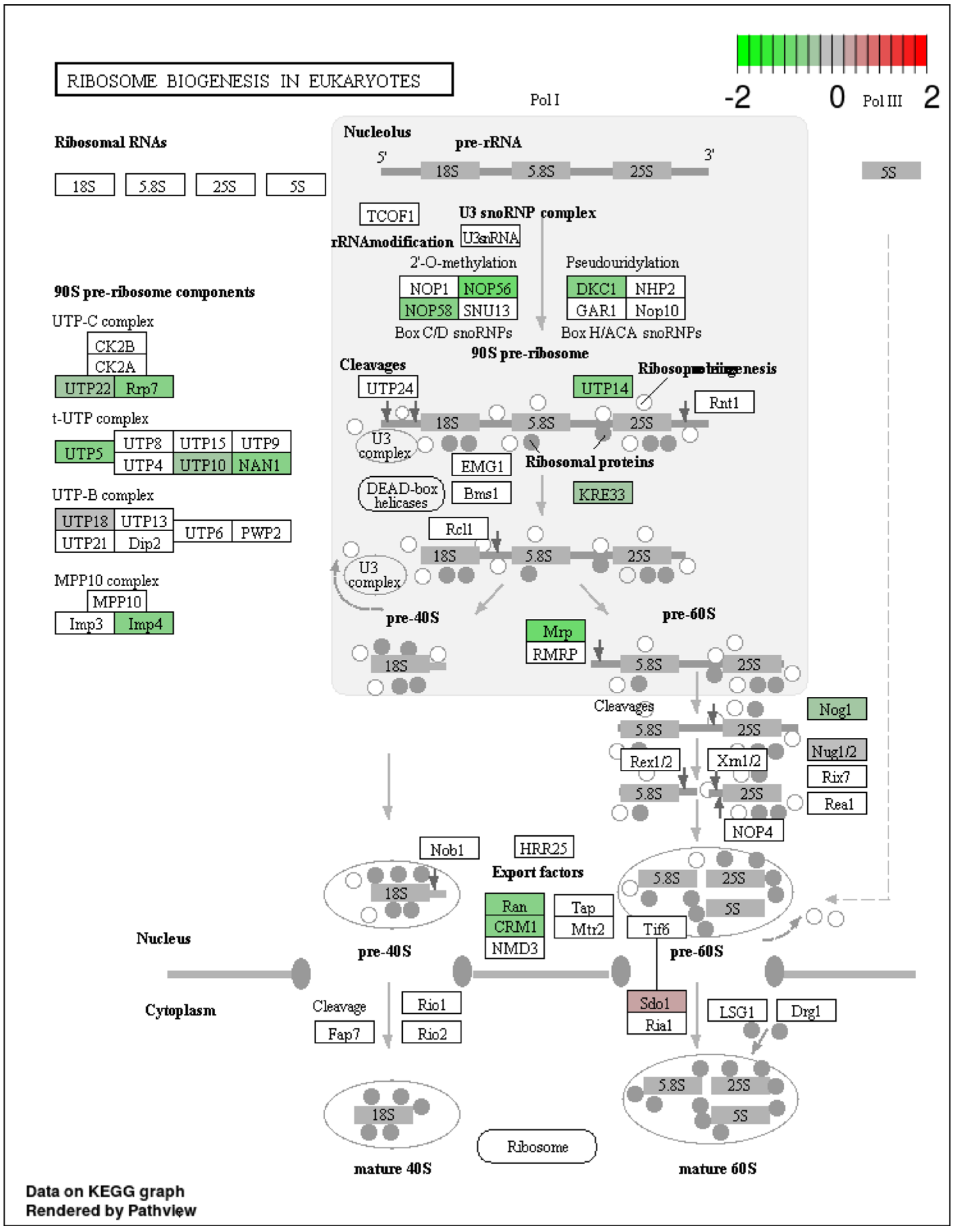

Supplementary Figure 9: USP46 and galeterone control ribosome biogenesis pathway. Ribosome biogeneisis KEGG pathway analysis comprised of genes regulated commonly by USP46 and galeterone.

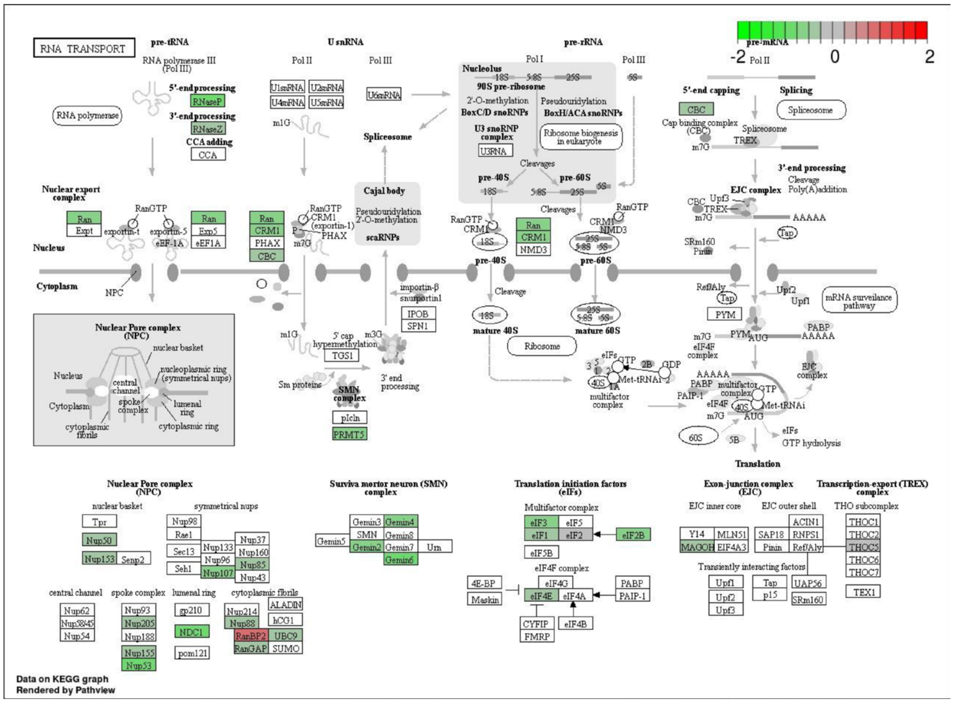

Supplementary Figure 10: USP46 and galeterone control RNA transport pathway. RNA transport KEGG pathway analysis comprised of genes regulated commonly by USP46 and galeterone.

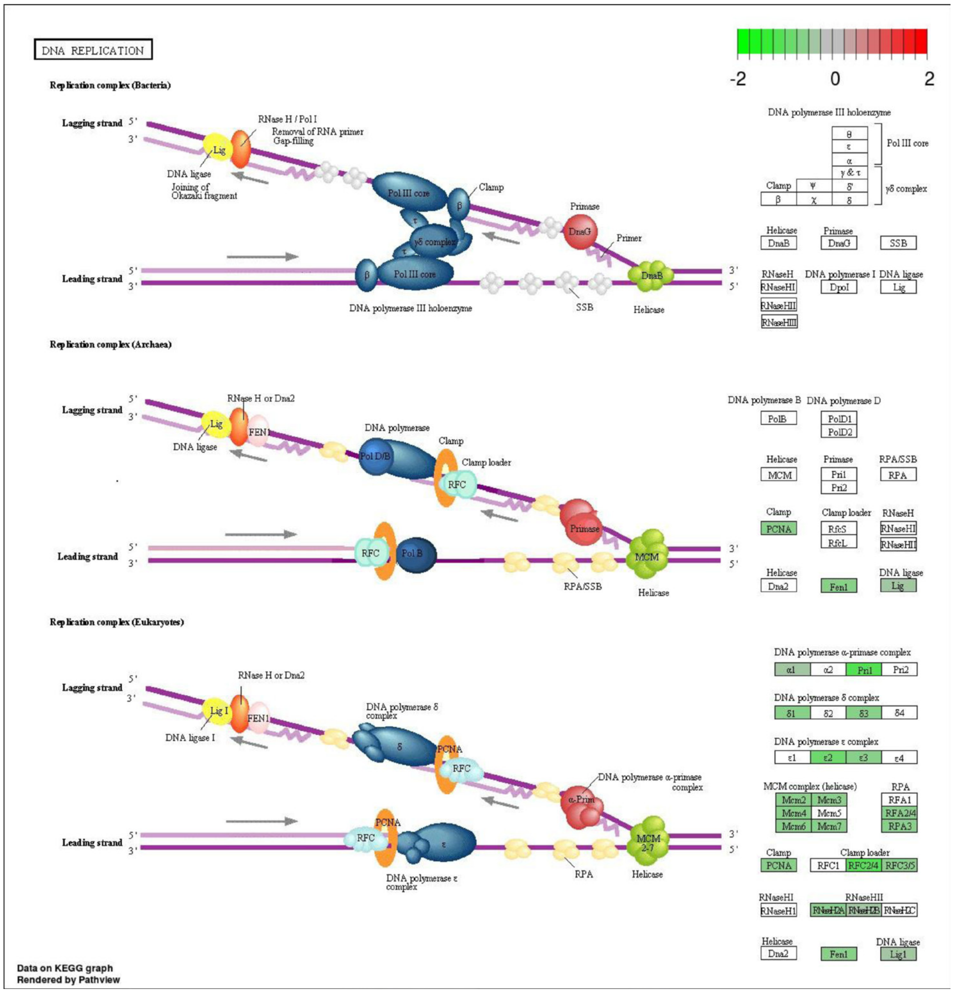

Supplementary Figure 11: USP46 and galeterone control DNA replication pathway. DNA replication KEGG pathway analysis comprised of genes regulated in the same fashion by USP46 and by galeterone.

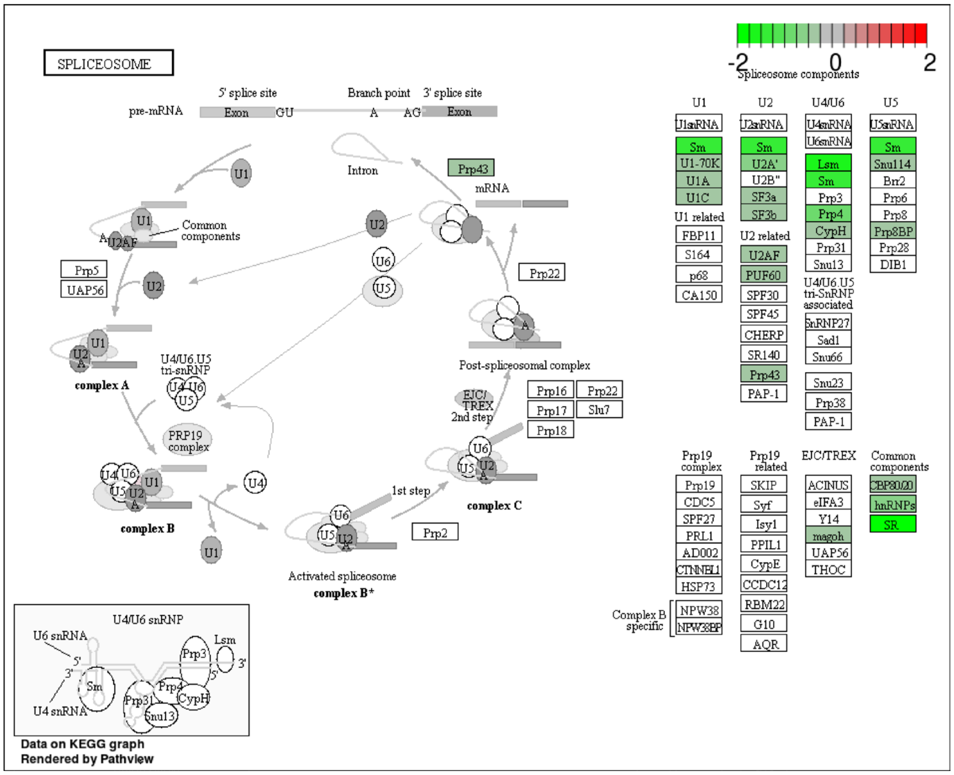

Supplementary Figure 12: USP46 and galeterone control spliceosome pathway. Spliceosome KEGG pathway analysis comprised of genes regulated in the same fashion by USP46 and by galeterone.

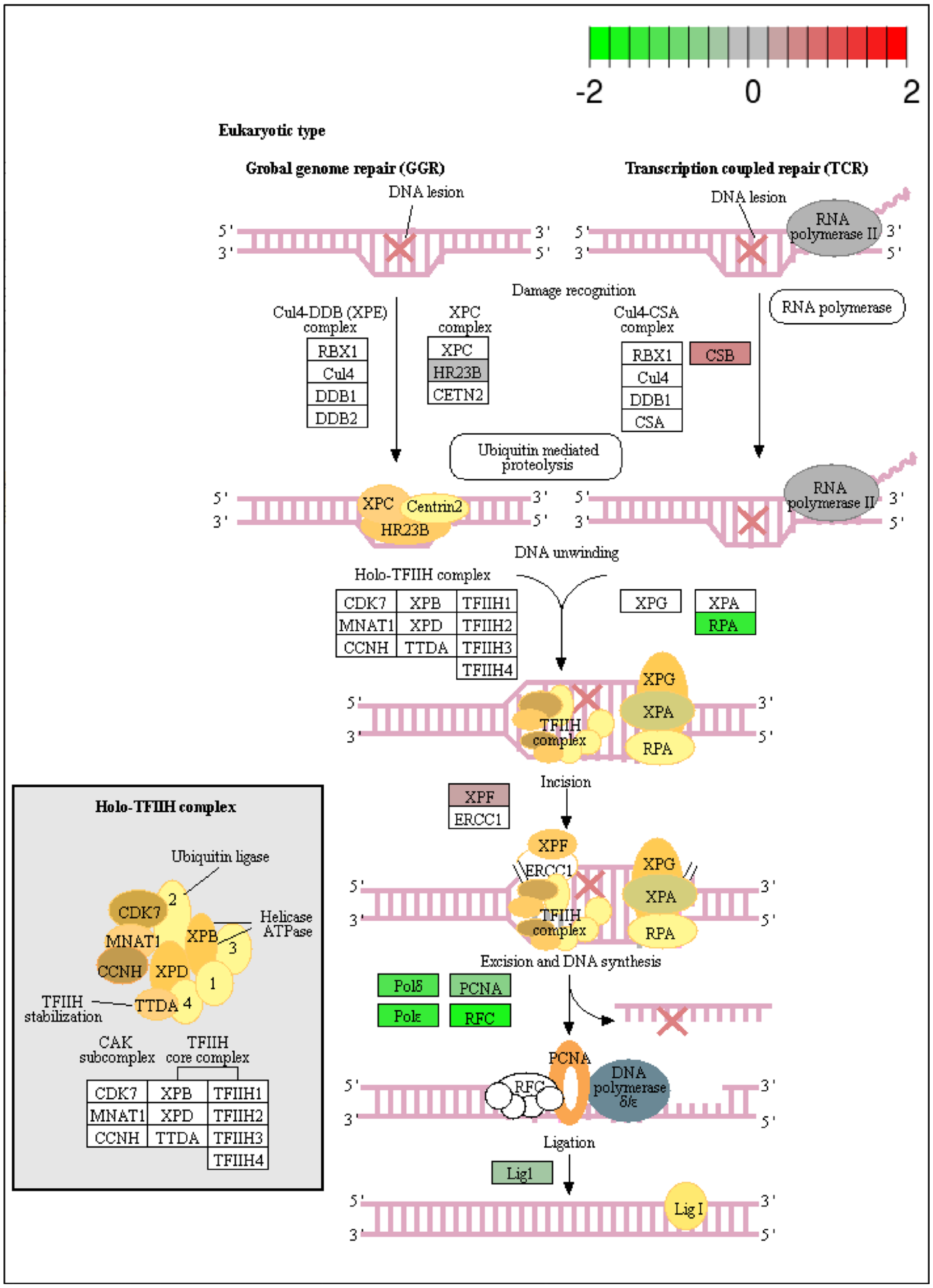

Supplementary Figure 13: USP46 and galeterone control nucleotide excision repair. Nucleotide excision repair KEGG pathway analysis comprised of genes regulated in the same fashion by USP46 and by galeterone.

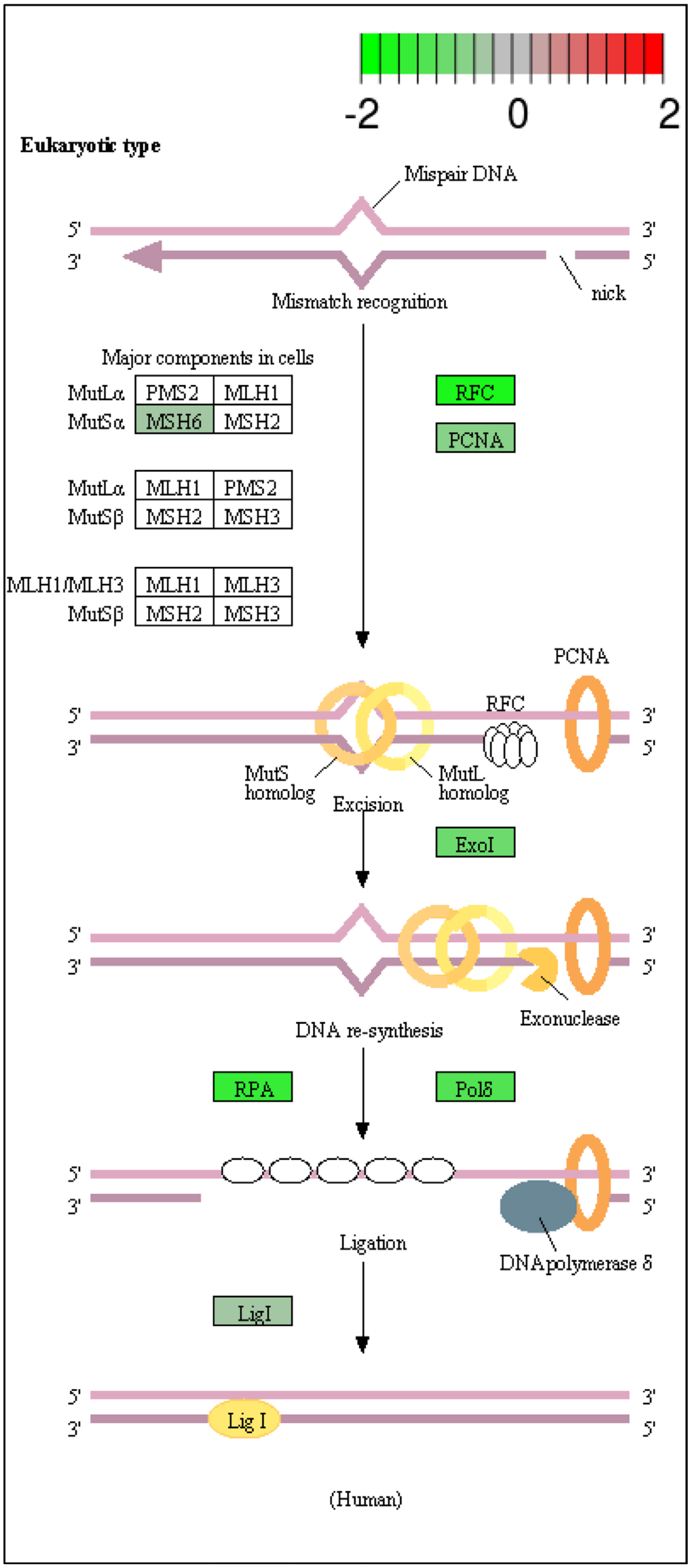

Supplementary Figure 14: USP46 and galeterone control mismatch repair pathway. Mismatch repair KEGG pathway analysis comprised of genes regulated in the same fashion by USP46 and by galeterone.

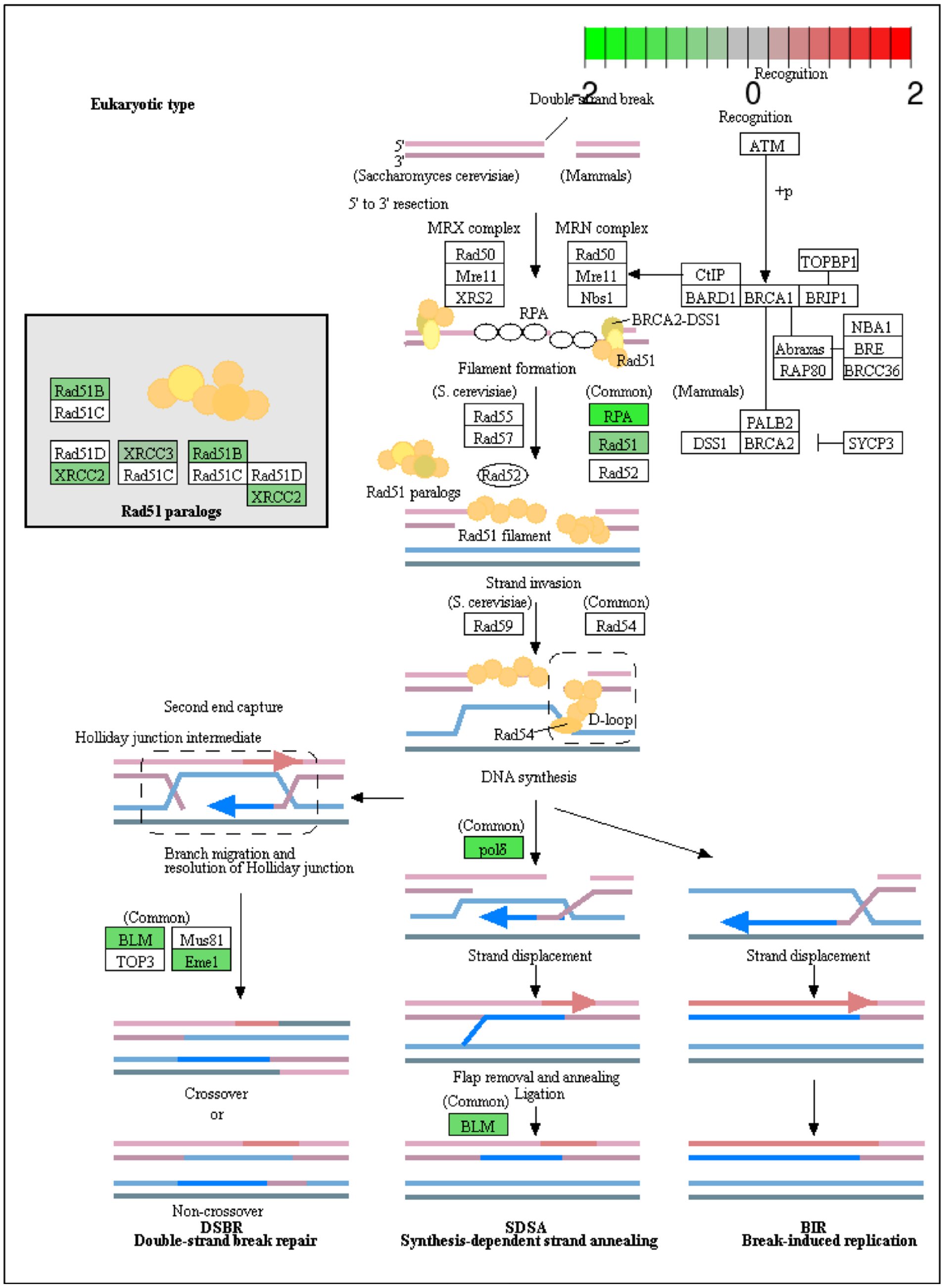

Supplementary Figure 15: USP46 and galeterone control homologous recombination pathway. Homologous recombination KEGG pathway analysis comprised of genes regulated in the same fashion by USP46 and by galeterone.

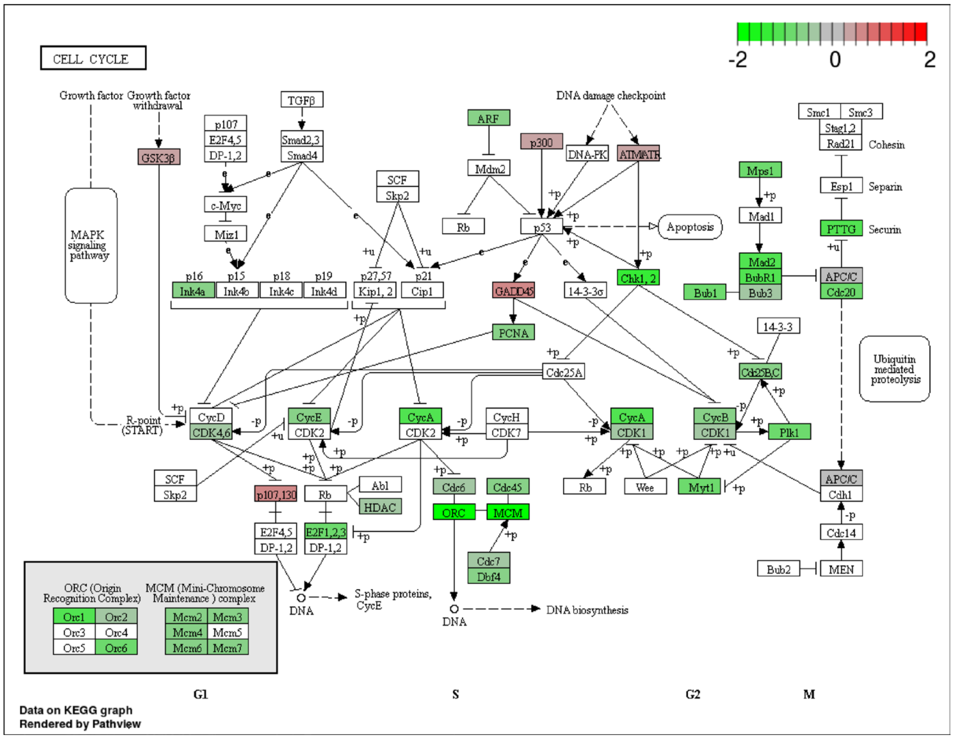

Supplementary Figure 16: USP46 and galeterone control cell cycle pathway. Cell cycle KEGG pathway analysis comprised of genes regulated in the same fashion by USP46 and by galeterone.

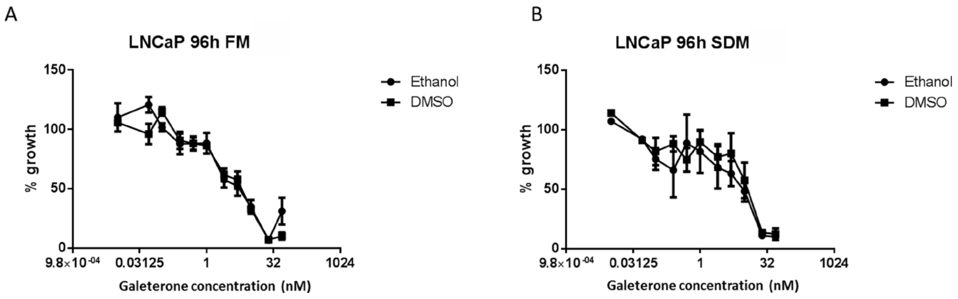

Supplementary Figure 17: Galeterone is equally effective in LNCaP cells in the presence and absence of androgen. LNCaP cells grown were seeded in full media (**A**) and in SDM media (**B**) after 24 h incubation cells were treated with varying concentrations of galeterone for 96 h and cellular proliferation was assessed by live cell imaging every 6 h with IncuCyte.

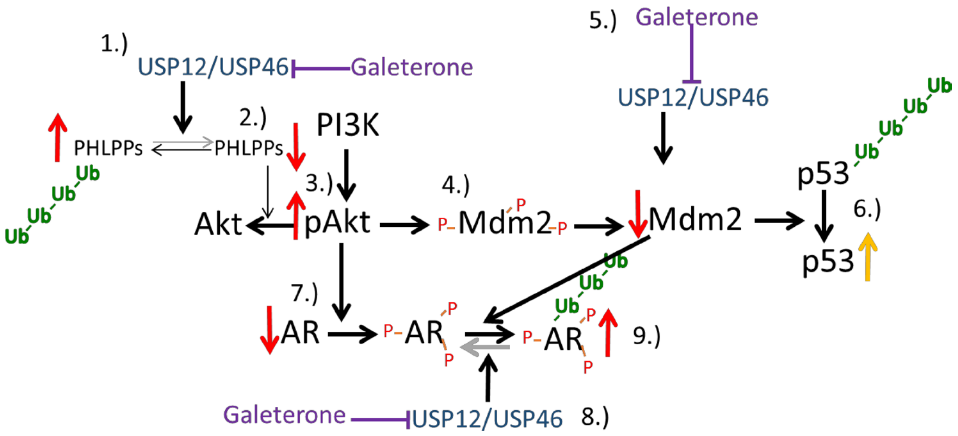

Supplementary Figure 18: Model of galeterone activity in PC. (1.) Galeterone inhibits USP12 and USP46 activity which prevents them from deubiquitinating the AKT phosphatases, PHLPP and PHLPPL [22, 21]. (2.) Decreased levels of PHLPPs lead to (3.) an increase in active pAKT [64] which (4.) in turn phosphorylates MDM2 [65]. (5.) Galeterone additionally inhibits USP12 and USP46 resulting in decreased MDM2 deubiquitination reducing MDM2 protein levels. (6.) This results in an increase in P53 tumour suppressor levels [66]. (7.) An increase in pAKT caused by galeterone also leads to AR phosphorylation at S213 and S791 which targets AR for ubiquitination by MDM2 and protein degradation [45]. (8.) At the same time galeterone inhibits USP12 and USP46 preventing them from deubiquitinating the AR [38]. (9.) This ultimately results in decreased AR protein levels (9.).
